# Deep Learning Empowers the Discovery of Self‐Assembling Peptides with Over 10 Trillion Sequences

**DOI:** 10.1002/advs.202301544

**Published:** 2023-09-25

**Authors:** Jiaqi Wang, Zihan Liu, Shuang Zhao, Tengyan Xu, Huaimin Wang, Stan Z. Li, Wenbin Li

**Affiliations:** ^1^ Research Center for Industries of the Future Westlake University Hangzhou 310030 China; ^2^ School of Engineering Westlake University Hangzhou 310030 China; ^3^ AI Lab Research Center for Industries of the Future Westlake University Hangzhou 310030 China; ^4^ Department of Chemistry School of Science Westlake University Hangzhou 310030 China; ^5^ Institute of Natural Sciences Westlake Institute for Advanced Study 18 Shilongshan Road Hangzhou Zhejiang Province 310024 China

**Keywords:** aggregation laws, deep learning, oligopeptides, self‐assembling

## Abstract

Self‐assembling of peptides is essential for a variety of biological and medical applications. However, it is challenging to investigate the self‐assembling properties of peptides within the complete sequence space due to the enormous sequence quantities. Here, it is demonstrated that a transformer‐based deep learning model is effective in predicting the aggregation propensity (AP) of peptide systems, even for decapeptide and mixed‐pentapeptide systems with over 10 trillion sequence quantities. Based on the predicted AP values, not only the aggregation laws for designing self‐assembling peptides are derived, but the transferability relation among the APs of pentapeptides, decapeptides, and mixed pentapeptides is also revealed, leading to discoveries of self‐assembling peptides by concatenating or mixing, as consolidated by experiments. This deep learning approach enables speedy, accurate, and thorough search and design of self‐assembling peptides within the complete sequence space of oligopeptides, advancing peptide science by inspiring new biological and medical applications.

## Graphical abstract

Peptide self‐assembly is essential for a variety of applications in biological and medical sciences. Transformer‐based deep learning has significantly expanded the explorable peptide sequence space to over tens of trillions of sequences. Design rules for achieving assemblies by mono‐component peptides, as well as by concatenation or mixing of peptides are elucidated, enabling fast, accurate, and thorough design.

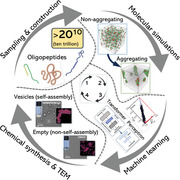

## 1 Introduction

Peptides, which are short chains consisting of normally less than 50 amino acids, have been paid tremendous attention due to their facile synthesis, inherent biodegradability, and biocompatibility, as well as rich chemical diversity for producing stable nanostructures with fluorescence, ^[^ ^1^ ^]^ semiconductivity,^[^ ^2^ ^]^ and piezoelectricity.^[^ ^3^, ^4^ ^]^ Albeit the extensive chemical diversity (> 20^50^), nowadays, only an infinitesimal portion of peptides have been found to self‐assemble into ordered structures,^[^ ^5^, ^6^, ^7^, ^8^ ^]^ due to the lack of efficient approaches for predicting their self‐assembling propensity, which is a prerequisite for producing desirable supramolecular morphologies and subsequent industrial applications (e.g., tissue engineering, drug delivery, and biosensing).^[^ ^9^ ^]^ In addition, self‐assembling of peptides has been widely involved in certain biological processes^[^ ^10^, ^11^, ^12^ ^]^(e.g., cell extension and contraction, movement of endocytic vesicles, intercellular transport of bacterial and viral pathogen) and protein misfolding diseases^[^ ^13^, ^14^, ^15^ ^]^ (e.g., Alzheimer's disease, Parkinson's disease, Type II diabetes). Therefore, elucidating the relationship between the position/type of constituent amino acids and self‐assembling of peptides is vital for inspiring new applications and unraveling the mysteries of various biological processes and diseases.

Over the past decades, self‐assembling peptides have been mostly discovered by human expertise combining wet‐lab experiments and inspired by biological systems. For instance, KLVFF^[^ ^16^ ^]^ and NFGAIL^[^ ^17^ ^]^ are derived from fragments of amyloid protein Aβ_16‐20_ and human islet amyloid polypeptide, respectively. However, experimentation normally spans a long period of time, and human expertise is often biased toward high *β*‐sheet propensity amino acids with moderate to high hydrophobicity,^[^ ^18^ ^]^ preventing a systematic and thorough search of the enormous sequence space of peptides. Therefore, an efficient and unbiased approach to discovering self‐assembling peptides is imperative.

**Figure 1:**
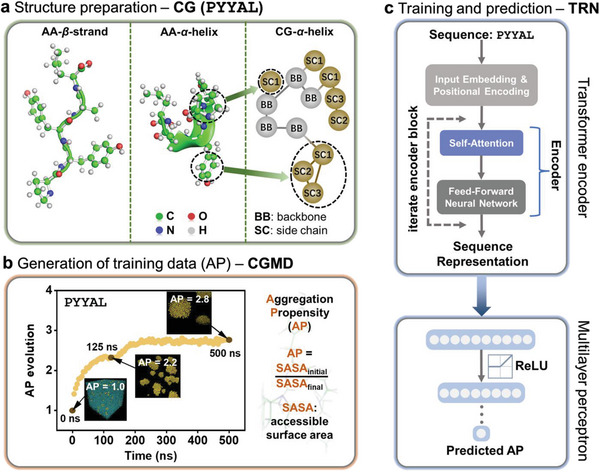
Workflow of coupled CGMD‐deep learning approach for effective discovery of self‐assembling oligopeptides. a) All‐atom (AA) models with *β*‐strand and *α*‐helix conformation, and coarse‐grained (CG) model with *α*‐helix conformation (used in this work), of an example pentapeptide PYYAL. The CG model approximates 3–5, mostly 4 atoms into one bead, aiming to accelerate simulation, as illustrated in dashed circles with BB representing a backbone bead and SC representing a side chain bead (Table S1, Supporting Information). b) Generation of aggregation propensity (AP) as training data by coarse‐grained molecular dynamics (CGMD). AP is defined as the ratio of accessible surface area at the beginning (SASA_initial_) and end (SASA_final_) of a CGMD simulation. An equilibration time of 125 ns is sufficient for achieving a reasonable AP (i.e., close to convergence), and it is therefore chosen as a trade‐off between simulation cost and accuracy. c) Architecture of transformer‐based regression network, comprising two parts, i.e., Transformer encoder and Multilayer Perceptron decoder, for extracting sequence representation and predicting AP, respectively.

In recent years, computational screening has been adopted for designing self‐assembling peptides. In 2015, Frederix et al.^[^ ^19^ ^]^ screened the aggregation propensity (AP, a prerequisite of self‐assembling; the definition of AP is given in **Figure** **1**a and also in Methods) of the complete sequence space of tripeptide employing coarse‐grained molecular dynamics (CGMD) and generated design rules for promoting self‐assembly. However, whether the design rules are transferable to peptides with more than three amino acids is dubious. CGMD also has its own limitation: as the length of peptides increases, the total number of peptides in sequence space will increase exponentially. Consequently, it will be an extremely expensive and intractable task to simply implement CGMD to thoroughly screen the sequence space of peptides containing more than four amino acids (which contain more than 20^[^ ^4^ ^]^ sequence quantities, e.g., 3.2 million for pentapeptides).

To overcome these challenges, emerging techniques employing artificial intelligence (AI) come into use.^[^ ^20^, ^21^, ^22^, ^23^, ^24^ ^]^ Batra et al.^[^ ^18^ ^]^ adopted an AI approach to predict the AP of pentapeptides based on the training data generated by CGMD, using the algorithm of random forest and Monte Carlo tree search. In this coupled CGMD‐AI approach, the quantity of training dataset is critical for effective training of the AI models, especially for high‐dimensional input data space distributing over a complex manifold.^[^ ^25^ ^]^ It can be assumed that for decapeptides and mixed pentapeptide systems with sequence quantity over one trillion, one would require much more data for effective training than that of pentapeptides with 3.2 million sequence quantities. However, it is expensive and time‐consuming to perform brute‐force CGMD simulations to generate a huge quantity of training data. Consequently, the traditional machine learning approaches (random forest, support vector machine, etc.) would gradually lose their capability, similar to the challenge encountered in brute‐force CGMD. Therefore, to the best of the authors’ knowledge, investigation of the sequence space of peptides containing more than five amino acids has never been reported.

Aiming to further push the limit of AP prediction to oligopeptide systems with an enormous sequence space (>10 trillion), we adopt a coupled CGMD‐deep learning approach, with CGMD for generating the training data of AP (Figure 1a,b) and the deep‐learning algorithm of Transformer‐based regression network (TRN) for AP prediction (Figure 1c). This Transformer architecture has been validated for its effectiveness on various sequence data, such as in natural language processing^[^ ^26^ ^]^ and protein structure prediction.^[^ ^27^, ^28^ ^]^ TRN employs a self‐attention module in Transformer^[^ ^29^ ^]^ to extract sequence representations and a nonlinear Multilayer Perceptron (MLP) for accurate prediction of AP. Compared to recurrent models such as Recurrent Neural Network^[^ ^30^ ^]^ and Long Short‐Term Memory,^[^ ^31^ ^]^ Transformer^[^ ^29^ ^]^ is more effective in capturing long‐range semantic associations, which renders it superior when applied to longer peptide sequences.

After testing the effects of secondary structure and simulation duration on aggregation (see Methods and Supplementary Materials SD1), we have demonstrated the prediction capability of TRN in pentapeptides, decapeptides, and mixed pentapeptide systems. Furthermore, we deduce the aggregation laws of pentapeptides with respect to the type and position of 20 natural amino acids. Last, but not the least, the transferability relation among the APs of pentapeptides, decapeptides, and mixed pentapeptide systems is revealed, inspiring the design of self‐assembling systems through the concatenation or mixing of peptides. It should be noted that in addition to AP, the morphology of aggregates is also critical in determining the properties of the resulting peptide‐based materials, which will be investigated in the near future but not included in this research.

## 2 Results and Discussion

### 2.1 Performance of AI Models

**Figure 2:**
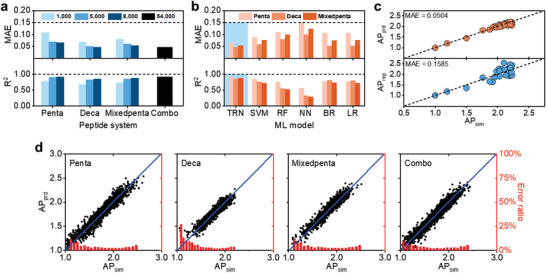
Performance of deep‐learning and non‐deep‐learning models. a) Performance of deep‐learning TRN models trained with 1000, 5000, and 8000 data and Combo model trained with 54 000 data. b) Performance comparison between deep‐learning TRN model and non‐deep learning models of support vector machine (SVM), random forest (RF), nearest neighbor (NN), Bayesian ridge (BR), and linear regression (LR), trained with 8000 data, for pentapeptides (Penta), decapeptides (Deca), and mixed pentapeptide (Mixedpenta) systems. c) Comparison between CGMD‐simulated AP (AP_sim_) and predicted AP (AP_prd_) by the Combo model, as well as AP_sim_ and reported AP (AP_rep_). A mean absolute error (MAE) of 0.0504 and 0.1585 is achieved for AP_sim_ versus AP_prd_, and AP_sim_ versus AP_rep_, respectively. d) Correlation and error ratio between AP_prd_ and AP_sim_. AP_prd_ is predicted by TRN models trained with 8000 data for pentapeptides, decapeptides, and mixed pentapeptides, and with 54 000 data for the Combo model.

During model training, we identify the effect of the number of training data on model performance (**Figure** **2**a) and illustrate the superiority of the deep learning algorithm of TRN to five non‐deep learning algorithms including support vector machine (SVM),^[^ ^32^ ^]^ random forest (RF),^[^ ^33^ ^]^ nearest neighbor (NN),^[^ ^34^ ^]^ Bayesian ridge (BR),^[^ ^35^ ^]^ and linear regression (LR)^[^ ^36^ ^]^ (Figure 2b). The mean absolute error (MAE) and coefficient of determination^[^ ^35^ ^]^ (*R*2) are adopted for assessing the performance of AI models, which are averaged results over ten parallel experiments (the exact values of MAE and *R*2 are shown in Table S2, Supporting Information).

As expected, the performance of TRN models improves as the size of the training dataset increases (Figure 2a), and notably, the TRN has achieved an *R*2 over 0.85 with only 8000 training data for the sequence space of decapeptide with over 10 trillion possibilities, proving the effectiveness of TRN in predicting the self‐assembly properties of peptides with enormous sequence space. With the same number of training data, the model performance of decapeptides and mixed pentapeptides is slightly reduced compared to that of pentapeptides (less than 8% with 8000 training data), which is intrinsically caused by the exponentially increased complexity of the sequence space of these two systems. In addition to separate models, we also train a model using combined AP data (#54 000 in quantity) of pentapeptides to decapeptides (i.e., penta‐, hexa‐, hepta‐, octa‐, nona‐, and decapeptides), termed as Combo model. It achieves the optimal performance with an MAE of 0.05 and *R*2 of 0.92, capable of predicting the AP of the complete sequence space of oligopeptides regardless of the peptide length.

The deep learning algorithm of TRN exhibits superiority to all five non‐deep learning algorithms (Figure 2b). For instance, the *R*2 of TRN trained with 8000 data for decapeptide is 0.85, while the counterpart of SVM and RF is 0.76 (TRN achieves 11.8% improvement) and 0.55 (TRN achieves 54.5% improvement), respectively. This indicates that TRN requires much less training data to reach a satisfactory level of accuracy than the non‐deep learning algorithms, holding promises for predicting the AP of polypeptides and proteins.

Comparing the CGMD‐simulated AP (AP_sim_) to deep‐learning predicted AP (AP_prd_) by Combo model as well as to the reported AP (AP_rep_) values of 26 pentapeptides that have been experimentally and computationally studied by Batra et al.^[^ ^18^ ^]^ (associated details can be found in Table SD1, Supporting Information) but never been involved in our model training or testing, the AP_sim_ versus AP_prd_ and AP_sim_ versus AP_rep_ are all in reasonable agreement, with an MAE of 0.0504 and 0.1585, respectively (Figure 2c), demonstrating the accuracy of our predictions with respect to ground‐truth CGMD simulations.

The error of AP_prd_ mainly lies at two ends of AP_sim_ distribution, especially at the low AP_sim_ range due to scarce sampling (Figure 2d). As AP_sim_ increases to 1.5, the error ratio (|AP_prd_‐AP_sim_|/AP_sim_×100%) remains ≈5%. The limited reliability of AP_prd_ at a low AP_sim_ range would have minimal effect on the selection of self‐assembling peptides mostly with medium to high AP values (AP > 1.5).

### 2.2 Effect of Hydrophilicity on Aggregation

**Figure 3:**
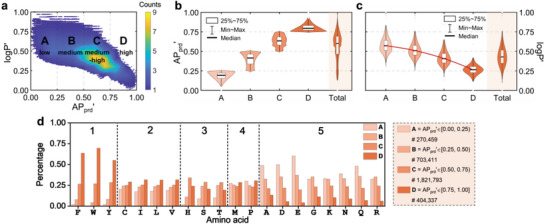
Aggregation laws of pentapeptides. a) Relation between logP' and AP_prd_' within the complete sequence space of pentapeptides (3.2 million in quantity). The AP_prd_' is divided into four ranges as low‐A, medium‐B, medium‐high‐C, and high‐D for more convenient and detailed analysis, i.e., A = AP_prd_'∈ [0.00, 0.25), B = AP_prd_'∈ [0.25, 0.50), C = AP_prd_'∈ [0.50, 0.75), and D = AP_prd_'∈ [0.75, 1.00]. The number of pentapeptides in each range is 270459, 703411, 1821793, and 404337. Color of blue to yellow indicates the number density. b,c) Violin distribution of AP_prd_' and logP' within four ranges of AP_prd_'. d) Percentage of amino acids summed over five positions (It should be noted that the term “five positions” should not be confused with the group number 1–5), within four AP_prd_' ranges.

It has been found that, in addition to AP, hydrophilicity (logP) also plays a crucial role in peptide aggregation and hydrogel formation.^[^ ^37^ ^]^ We hereby present a complete picture of AP_prd_' versus logP' (the prime symbol ‘ denotes normalization between 0 and 1) for pentapeptides with 3.2 million sequence possibilities, as shown in **Figure** **3**a–c. Among 3.2 million pentapeptides, more than 2.9 million (>90%) exhibit medium to high AP_prd_’ (range B to C, Figure 3a), suggesting significant potential for achieving aggregates and self‐assemblies. As AP_prd_' increases from low to high (range A to D, Figure 3a), the median value of logP' consecutively decreases (Figure 3c), indicating that aggregates tend to form with stronger hydrophobicity. However, intermediately hydrophilic or amphiphilic pentapeptides (logP' ∈ [0.25, 0.75]) exhibit a wide range of AP_prd_' from 0 to 1 (Figure 3a,c), similar to observations in tripeptides.^[^ ^19^ ^]^ In summary, AP_prd_' only exhibits a weak correlation with logP', evidencing that hydrophobicity is not the sole contributor to aggregation and highlighting the importance of unbiased screening through CGMD‐AI without assumptions of aggregating mechanisms.

To account for the influence of both AP and logP in the self‐assembly of peptides, a hydrophilicity‐corrected score AP_H_^[^ ^19^ ^]^ has been developed to introduce bias to soluble peptides (we use AP_H_^2‐0.5^ to denote the parameters in our AP_H_ calculation. See Equation 2 in Experimental Section). In addition, in this work, we propose another optimized score AP_HC_ by penalizing the contribution of logP at both soluble and insoluble ranges (Equation 4; SD2, Supporting Information). The relations of AP_H_^2‐0.5^′‐ logP' and AP_HC_'‐ logP' are presented in Figures S1a–c,S2a–c (Supporting Information). The corrected scores (AP_H_^2‐0.5^′ and AP_HC_') significantly penalize AP_prd_' within the high hydrophobicity range (range D, Figure 3a) and favor peptides with medium hydrophilicity (range H in Figure S1a–c and range M in Figure S2a–c, Supporting Information). This indicates that the corrected scores AP_H_^2‐0.5^′ and AP_HC_' are capable of distinguishing between aggregation and precipitation, beneficial for selecting self‐assembling peptide candidates for hydrogel formation. Comparing AP_H_^2‐0.5^′ and AP_HC_', the former assigns a high score to peptides mostly with logP' close to 0.25 (range H, Figure S1c, Supporting Information), while the latter assigns high scores to peptides with logP' close to 0.5 (range M, Figure S2c, Supporting Information), better matching the distribution of logP' among aggregating peptides in experiment (Figure SD5a, Supporting Information). In summary, AP_HC_' shows improved accuracy over the existing score of AP_H_^2‐0.5^′, further minimizing the self‐assembling peptides library by reducing bias to both hydrophilic and hydrophobic peptides.

### 2.3 Aggregation Laws

In order to derive aggregation laws, i.e., the effects of position and type of amino acids on peptide aggregation, we analyze the total percentage of the 20 amino acids summed over five positions, as well as the percentage in each position, within four ranges of AP_prd_' (Figure 3d; Figure S3, Supporting Information), AP_H_^2‐0.5^′ (Figures S1d,S4, Supporting Information), and AP_HC_' (Figures S2d,S5, Supporting Information). Based on their contribution to AP_prd_', these 20 amino acids are divided into five groups, each indicated by an Arabic number between 1 and 5 (Figure 3d). Regarding AP_H_^2‐0.5^′ and AP_HC_', we use the same grouping approach for the convenience of direct comparison.

Group 1 contains aromatic amino acids F, Y, and W (Group 1, Figure 3d), which contributes most to aggregation through π‐stacking^[^ ^38^ ^]^ (an example is shown in Figure S6a, Supporting Information) and cooperatively hydrophobicity. Comparing the percentage of F, Y, and W calculated based on AP_H_^2‐0.5^′ and AP_HC_' to that of AP_prd_', the contribution of W has been brought down at a high range (Group 1, Figures S1d,S2d, Supporting Information) due to stronger hydrophobicity. This is consistent with our experimental findings that the pentapeptide WWWWW precipitates in water solvent at 25 mm forming suspension, while FFFFF and YYYYY form hydrogels with nanosheet and nanofiber structures(Figure S7, Supporting Information). F, Y, and W are found favorable in positions 3–5 (especially 3rd position) for promoting self‐assembling (range D, H, and M in Group 1, Figures S3–S5, Supporting Information, respectively). We propose that at the third position, the aromatic amino acids should have more degree of freedom to drive self‐assembling by *π–π* interactions. When the aromatic amino acids are located at the C‐terminus (second favored position), they act as strong hydrogen acceptors (preset in Martini2 force field, see Table S1, Supporting Information) and would possibly form specific structures with other peptides in the solvent, increasing the distance between the benzene rings and thus reduce the π‐π interactions. The aromatic residues act as both hydrogen acceptor and donor when they are located in the third position and becomes more challenging to interact with other peptides through hydrogen bonding due to steric hindrance, and therefore no specific structures can be formed, but the benzene rings will then have more freedom to interact with each other and promote aggregations.

In Group 2, I, L, and V carry hydrophobic sides, and C shares a structure with the amino acid V carrying a ‐SH group (Group 2, Figure 3d). C, I, L, and V promote aggregation due to the hydrophobic repulsion existing between side chains and water (Figure S6b, Supporting Information). However, the percentage of I, L, and V contained in peptides within high‐score ranges of AP_H_^2‐0.5^′ (Group 2, Figure S1d, Supporting Information) and AP_HC_' (Group 2, Figure S2d, Supporting Information) has been penalized due to that peptides containing those amino acids tend to have high hydrophobicity. C, I, L, and V are mostly found occupying positions close to two termini of peptides, especially N‐terminus in aggregated peptides (range D, H, and M in Group 2, Figures S3–S5, Supporting Information).

H, S, and T amino acids (Group 3. Figure 3d) have polarizable side chains and thus H‐, S‐, and T‐contained peptide systems could promote aggregation through hydrogen bonding (a type of dipole force, Figure S6c, Supporting Information). However, within the high‐score range (Group 3, Figure 3d; Figures S1d,S2d, Supporting Information), the percentage of H, S, and T decreases mainly due to the fact that the dipole‐dipole interaction has a minor contribution than π‐stacking to aggregation. It is observed that S and T prefer to occupy N‐ and C‐termini, with the N‐terminus bearing a higher preference, while H tends to occupy positions away from two termini (range D, H, and M in Group 3, Figures S3–S5, Supporting Information). The exposure of S and T at two termini should be conducive to the formation of specific packing between peptides through hydrogen bonding, such as polar zippers^[^ ^39^ ^]^ between neighboring *β*‐sheets.

M and P amino acids are almost equally found across four AP_prd_' ranges, with a slight increase at high AP_prd_' range (Group 4, Figure 3d), while the AP_H_^2‐0.5^′ and AP_HC_' significantly increase the percentage of P at high range and decrease that of M (Group 4, Figures S1d,S2d, Supporting Information). P is found to favor position 1 and position 2 (range D, H, and M in Group 4, Figures S3–S5, Supporting Information), similar to the reported finding that P prefers position 1 in tripeptide for promoting aggregation,^[^ ^19^ ^]^ due to its unique kink conformation (Figure S6d, Supporting Information) allowing better packing of the short peptides.^[^ ^40^ ^]^

The amino acids in group 5 are ones not favorable for aggregation on a statistical level based on AP_prd_' value, which can also be divided into three subgroups: 1) negatively charged D and E, and positively charged K and R, 2) N and Q with strong polarity, 3) A and G with no side chain in coarse‐grained representation (Table S1, Supporting Information). Different from AP_prd_', AP_H_^2‐0.5^′ and AP_HC_' significantly increase the percentage of charged amino acids at the high range (Group 5, Figures S1d,S2d, Supporting Information). Negatively charged amino acids D and E are favored in positions close to C‐terminus, while positively charged R and K prefer positions close to N‐terminus for promoting aggregation (range D, H, and M in Group 5, Figures S3–S5, Supporting Information). D and E residues at C‐terminus (as well as R and K at N‐terminus) could produce a doubly charged head group and promote self‐assembly through the attraction of opposite charges and formation of salt bridges, driving intermolecular alignment. Examining the average distance between the negatively charged amino acid E and nearest neighbor N‐terminus (positively charged) of amino acid I with respect to the pentapeptide IPWCE (r_near_), the distance r_near_ (= 5.7 Å) in the aggregate is smaller than that of the pair in the peptide sequence itself (r_self_ = 6.4 Å), demonstrating that E attracts the N‐terminus of other peptides and forms salt bridges (Figure S6e, Supporting Information). This finding is also consistent with that of Frederix, et al.^[^ ^19^ ^]^ N and Q are barely found in high levels of AP_H_^2‐0.5^′ and AP_HC_' (Group 5, Figures S1d,S2d, Supporting Information), due to the strong hydrophilicity of those two amino acids prefer to induce solvation. Compared with other amino acids, A and G are not favored for the formation of aggregates due to the lack of dominating interactions.

### 2.4 Transferability of AP Values

Aiming to discover self‐assembling peptide systems through the concatenation or mixing of pentapeptides, we have studied the transferability relation among 1 million APs of decapeptides (AP_deca_), APs of mixed pentapeptide systems (AP_mixpen_), and AP of averaged pentapeptides (AP_avepen_) (Figure S8a,b, Supporting Information). To consolidate our findings here, we have also studied the transferability relation of 2253 groups of AP_avepen_ and AP_deca_ (AP_mixpen_), using CGMD‐generated data instead of predicted APs (Figure S9a,b, Supporting Information).

AP_avepen_ and AP_deca_ (AP_mixpen_) generally exhibit a linear correlation (Figure S8a,b, Supporting Information) with a mean absolute error of 0.077 (0.086), indicating that most decapeptides and pentapeptides (mixed pentapeptide systems) obey the same aggregation laws. Tthus the AP of concatenated or mixed systems can be roughly estimated by averaging the AP of the constituent peptides.

Examining the distribution of AP difference (AP_deca_‐AP_avepen_ and AP_mixpen_‐AP_avepen_, Figure S8c,d, Supporting Information), it is found that more than 94.2% AP_mixpen_ is larger than AP_avepen_ with a median value of 0.064 and a maximum AP difference of 0.549 and 0.634 is achieved regarding AP_deca_‐AP_avepen_ and AP_mixpen_‐AP_avepen_, respectively. In addition, more than 500 of AP_deca_‐AP_avepen_ and 2457 of AP_mixpen_‐AP_avepen_ are found larger than 0.4, proving that aggregation can be promoted by concatenating or mixing peptides. The AP_deca_‐AP_avepen_ and AP_mixpen_‐AP_avepen_ exhibit a positive correlation (Figure S8e, Supporting Information), demonstrating similar dominant mechanisms in promoting self‐assembly in decapeptides and mixed pentapeptide systems. We reach the same conclusions from the CGMD‐generated AP data (Figure S9, Supporting Information), proving the validity of the predicted results.

**Table** **1** lists five peptide groups with AP_deca_–AP_avepen_ larger than 0.4 and AP_deca_ larger than 1.5. For all those five decapeptides and mixed pentapeptides systems, they are all concatenated or mixed by corresponding pentapeptide subgroups (Pen1 and Pen2), and at least one of the corresponding pentapeptide subgroup does not exhibit strong aggregation propensity. Examining the sequence feature of five decapeptides and mixed pentapeptide systems, the two pentapeptide subgroups comprise different types of charge (blue: +e; yellow: −e). It is reasonable to deduce that the Coulombic attraction significantly promotes aggregation in such concatenated or mixed systems, possibly leading to ordered self‐assembled structures. Examining the sequences of the top 200 AP_mixpen_‐AP_avepen_ from the prediction (Table S3, Supporting Information) and the top 100 AP_mixpen_‐AP_avepen_ from CGMD simulation (Table S4, Supporting Information), the same mechanism (i.e., Columbic interaction) for promoting aggregation in decapeptides and mixed pentapeptide systems can be inferred.

**Table 1:** AP_prd_ and logP information of five groups of pentapeptides, decapeptides, and mixed pentapeptides, with AP_deca_ larger than 1.5 while AP_deca_‐AP_avepen_ larger than 0.4.

Pen1| Pen2	AP_pen1_	AP_pen2_	AP_avepen_	logP_pen1_	logP_pen2_	AP_deca_	AP_mixpen_	AP_avepen_‐AP_deca_	AP_avepen_‐AP_mixpen_
NRMMR| DMGID	1.150	1.144	1.147	0.474	0.597	1.659	1.612	−0.512	−0.465
DMTAL| IAGAK	1.155	1.031	1.093	0.451	0.498	1.542	1.366	−0.449	−0.273
RLNCK| ADMGE	1.167	1.125	1.146	0.511	0.653	1.591	1.606	−0.445	−0.460
LRLRL| IQDEC	1.480	1.103	1.292	0.360	0.606	1.731	1.735	−0.440	−0.443
IVNRR| EEEQS	1.174	1.035	1.105	0.466	0.788	1.531	1.490	−0.427	−0.385

**Figure 4:**
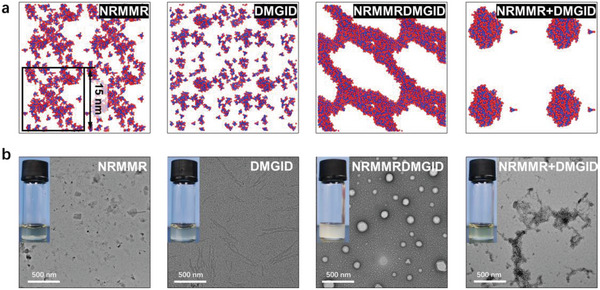
Computational and experimental morphologies of pentapeptides, decapeptides, and mixed pentapeptides. a) Computational morphologies of pentapeptides NRMMR, DMGID (simulated for 1.25 µs), decapeptide NRMMRDMGID (simulated for 6.25 µs), and mixed pentapeptide systems of NRMMR and DMGID (simulated for 1.25 µs). Color blue indicates backbone beads while red indicates side chain beads. b) TEM images with photographs of pentapeptides NRMMR, DMGID, decapeptide NRMMRDMGID, and mixed pentapeptide systems of NRMMR and DMGID in water solvent. The concentration of each peptide is 25 mmol L^−1^.

To consolidate the findings from the predictions, we perform 1.25 µs CGMD simulations on the aforementioned five groups of pentapeptides and mixed pentapeptides, and 6.25 µs simulations on decapeptides (**Figure** **4**a; Figure S10, Supporting Information), to achieve reliable morphologies by eliminating the non‐convergence effect (the morphologies simulated at 125 ns is shown in Figure S11, Supporting Information). The long‐duration CGMD simulation generates results consistent with the predictions that the subgroup pentapeptides do not exhibit a strong propensity for aggregation while the concatenated decapeptides and mixed pentapeptides demonstrate strong aggregation. In addition, wet‐lab experiments are performed on pentapeptides NRMMR and DMGID, decapeptide NRMMRDMGID, and mixed pentapeptide system NRMMR+DMGID, which are chemically synthesized at pH 7 and characterized by TEM after 48 h of standing, for examining the degree of aggregation as well as morphologies (Figure 4f). As the TEM images show, NRMMR and DMGID do not exhibit clear aggregation after 48 h, while NRMMRDMGID and NRMMR+DMGID exhibit vesicle and amorphous aggregated morphologies, respectively. This vesicle structure has also been directly observed in 6.25 µs CGMD simulations (third column Figure 4a), proving the predictive power of CGMD simulation in morphology as well. Vesicle structures can be employed for drug delivery in future development on medical applications, and similar vesicle structures could be possibly achieved by the sequence candidates of decapeptides provided (Tables S3,S4, Supporting Information).

## 3 Conclusion

With the approach of coupled CGMD and deep learning, we have successfully predicted the AP of pentapeptides, decapeptides, and mixed pentapeptides, enabling the derivation of aggregation laws and transferability study for accelerated discovery and design of self‐assembling peptide systems and possible applications.

The prediction accuracy of AI models trained with TRN all reaches an excellent level to above 85% with only 8000 training data, even with a sequence space of over 10 trillion possibilities, superior to traditional non‐deep learning algorithms (e.g., SVM, RF, NN, BR, and LR). A combo model trained with 54000 AP data achieves desirable accuracy (i.e., *R*2 = 0.92) and transferability in AP prediction (i.e., capable of predicting AP of any pentapeptide to decapeptide, regardless of peptide length).

The predicted AP provides a complete picture of the relation between AP and logP, manifesting that only a weak correlation exists between the two quantities, which illustrates the necessity of complete and unbiased AP prediction using AI. Based on the predicted AP values (AP_prd_) and hydrophilicity‐corrected AP_H_^2‐0.5^ and AP_HC_, aggregation laws regarding 20 natural amino acids are derived. For promoting aggregation, aromatic amino acids (F, W, and Y) and ones carrying hydrophobic side chains (I, L, and V) should be placed at positions close to the C‐terminus. In addition, negatively charged amino acids (D, E) and positively charged amino acids (R, K) prefer positions close to the C‐ and N‐terminus, respectively. The polar amino acids S and T can promote aggregation when located at N‐terminus. Amino acid C also plays an important role when located at two termini, and the contribution of P also stands out when located at N‐terminus.

The transferability relation between AP_avepen_ and AP_deca_ as well as between AP_avepen_ and AP_mixpen_ reveals that, while most decapeptides and mixed pentapeptides systems obey the same aggregation rules as mono‐component pentapeptide systems, the concatenation or mixing of pentapeptides could produce a new possibility for achieving self‐assemblies due to the Columbic interactions that exist between the pentapeptide subgroups, as confirmed by both simulation and experiments.

The above findings advance peptide science by demonstrating the effectiveness of the TRN network in predicting the enormous sequence space of oligopeptides, and by discovering the aggregation laws and transferability relation between peptide subgroups and concatenated/mixed peptide systems. The discovery of self‐assembling peptides/peptide mixtures with sequence quantities over 10 trillion is significantly accelerated, expediting the development of biological and medical applications that employs short peptide building blocks as key structural and functional elements.

## 4 Experimental Section

### Evaluation of Secondary Structure and Simulation Duration

Previous works assumed identical conformation (i.e., *β*‐strand) for every peptide in simulations ^[^ ^18^, ^19^ ^]^ ，while longer peptides such as decapeptides might adopt different secondary structures and exert influence on aggregation. Therefore, the total energy of a single pentapeptide/decapeptide with *β*‐strand/*α*‐helix conformation in water at 300 K was first evaluated. The total energy was a sum of bonded (i.e., bond, dihedral, proper, and improper interactions) and non‐bonded interactions (Lennard–Jones and Columbic interactions) while excluding kinetic energy. *β*‐strand and *α*‐helix were the two most common secondary structures, and the Ramachandran angles^[^ ^41^ ^]^ of each were set as *ϕ* = −119°, *ψ* = 113° and *ϕ* = −57°, and *ψ* = −47°, respectively, which are the dominant Ramachandran angles found in experiments.^[^ ^42^ ^]^ Forty thousand parallel coarse‐grained molecular dynamics (CGMD) simulations at 300 K for 5 00 000 steps with a time step of 25 fs (i.e., 12.5 ns) were conducted, each containing one *β*‐strand/*α*‐helix pentapeptide/decapeptide solvated in a 5 nm box containing 1045 water beads. The CGMD simulation was performed with the open‐source GROMACS package (version 5.1.5)^[^ ^43^ ^]^ and Martini force field (version 2.2).^[^ ^44^, ^45^, ^46^ ^]^ The total energy was output every 100 steps, and the final total energy for each peptide was obtained through averaging over the last 2 50 000 steps (from 6.25 to 12.5 ns, Figure SD1, Supporting Information). The relevant discussion of the effect of secondary structure as well as simulation duration is included in SD1 (Supporting Information).

### Generation of the AP Training Data with CGMD

First, Latin Hypercube sampling^[^ ^47^ ^]^ was adopted to sample 8000 sequences from the complete sequence space of pentapeptide, aiming to collect uniform training samples. The selected peptide sequences were then fed to CGMD simulations for the generation of AP, which were performed with the open‐source package GROMACS^[^ ^43^ ^]^ and Martini force field version 2.2.^[^ ^44^, ^45^, ^46^ ^]^ This Martini force field had been widely adopted in the study of peptide/protein self‐assembling and the resulting new physics suggested had been confirmed by experiments.^[^ ^18^, ^19^, ^48^, ^49^, ^50^, ^51^ ^]^ It was thus expected that the level of aggregation could be well represented in CGMD simulations.

The all‐atom pentapeptide structures were prepared based on CHARMM36,^[^ ^52^ ^]^ and then coarse‐grained using the Python script martinize.py.^[^ ^44^, ^45^ ^]^ In total, 150 coarse‐grained pentapeptides (or 81 decapeptides) were solvated randomly in a 15 nm × 15 nm × 15 nm box with 28 400 water beads (water density ≈1 g cm^−3^), resulting in a solvent concentration of 0.074 mol L^−1^ for pentapeptides (0.040 mol L^−1^ for decapeptides), close to those in the experiment. The charge of the solution was maintained neutral by adding a proper amount of Na^+^ or Cl^−^. The whole system was then energy‐minimized using the steepest descent algorithm,^[^ ^53^ ^]^ until the maximum force on each atom was less than 20 kJ mol^−1^ nm^−1^. Subsequently, the system was passed to an equilibration run for 5 × 10^6^ steps, with a time step of 25 fs, resulting in a total simulation time of 125 ns (or “effective time” 600 ns). The temperature and pressure during the equilibration were controlled through the Berendsen algorithm at 300 K and 1 bar, respectively. The AP value was calculated at the last step (see Scoring methods). In AP transferability study between pentapeptides, decapeptides, and mixed pentapeptides, 1.25 µs (or “effective time” 6 µs due to the acceleration of coarse‐graining of four atoms into one bead) for the selected five groups of pentapeptides and mixed pentapeptides and 6.25 µs (or “effective time” 25 µs) for the decapeptides and for achieving reliable morphologies, were run.

### Scoring Methods

It was declared that there were three types of AP values in this work, which were AI predicted value AP_prd_, hydrophilicity‐corrected score AP_H_^2‐0.5^, and corrected AP_H_^2‐0.5^ score AP_HC_ (see details in SD2, Supporting Information). Their normalized counterparts (between 0 and 1) AP_prd_', AP_H_^2‐0.5^′, and AP_HC_' were utilized for analysis throughout the Main Body.

The AP is defined as the ratio between the accessible surface areas at the beginning (SASA_initial_) and end of (SASA_final_) a CGMD simulation,^[^ ^54^ ^]^ as shown in Equation 1:

(1) $$\mathrm{AP}=\frac{{\mathrm{SASA}}_{\mathrm{initial}}}{{\mathrm{SASA}}_{\mathrm{final}}}$$

Based on the CGMD‐generated AP and AI model, the AP values of remaining pentapeptides were predicted, thus AP_prd_ shared the same meaning as AP.

The normalized AP_prd_ (i.e., AP_prd_') and hydrophilicity logP (i.e., logP') were then employed to calculate the hydrophilicity‐corrected score AP_H_^2‐0.5^ by^[^ ^19^ ^]^:

(2) $${\mathrm{AP}}_{H}^{2-0.5}=AP_{\mathrm{prd}}{{}^{\prime }}^{2}\times \log{P^{\prime }}^{0.5}$$

The logP is defined as the sum of the Wimley–White whole‐residue hydrophilicities,^[^ ^55^ ^]^ as in Equation 3:

(3) $$\mathrm{logP}=\sum_{i\;=\;1}^{n}\Delta G_{\mathrm{woct},i}$$

Here, *n* denotes the number of residues in one peptide (for example, *n* equals to 5 for pentapeptides). Δ*G*_woct,i_ (in kcal mol^−1^) represents the free energy of transfer from water to *n*‐octanol for the *i*‐th residue.

To alleviate the bias of the score function AP_H_^2‐0.5^ to soluble (as well as insoluble) peptides,^[^ ^19^ ^]^ a penalty factor acting on logP' is included, as defined by:

(4) $${\mathrm{AP}}_{\mathrm{HC}}={\mathrm{AP}}_{H}^{2-0.5}\times e^{-\frac{{\left(\log P^{\prime }-\mu \right)}^{2}}{2\sigma^{2}}}$$

Here, *µ* (= 0.4113) and *σ^2^* (= 0.0657) are the average and variance of the logP' of aggregated peptides among the 12 pentapeptides which have been experimentally validated for aggregation (Table SD1, Supporting Information).

### AI Model Training

The AP predictive models were trained with a Transformer‐based regression network, which consisted of a Transformer encoder and a downstream Multilayer Perceptron (MLP) decoder (Figure 1c). The transformer encoder was decomposed into two parts: input embedding and positional encoding and encoder block. Input embedding mapped discrete words (i.e., amino acids) in a peptide sequence into a continuous space of 512 dimensions. Compared with one‐hot encoding, the word embedding was capable of learning the embedding of each amino acid based on the variability between amino acids, contributing to more accurate predictions. The positional encoding embedded the position information of words into the outputs of input embedding. The encoder block contained a self‐attention network and a feed‐forward neural network, each containing a residual connection and a layer normalization. The number of heads was set as 8, and the dimensions of K, Q, and V as 64 (hyperparameters of the self‐attention module). The dimension of hidden layers in a feed‐forward neural network was set to 2048. There were a total of 6 encoder blocks concatenated to perform the extraction of sequence representations.

The transformer encoder outputs a hidden layer representation of the peptide sequence, which was dimensionally reduced five times in the MLP as [512, 256, 64, 32, and 1] after being compressed into a 1D vector. Each MLP layer contained a linear layer responsible for the dimensionality reduction mapping and a nonlinear activation function of Leaky ReLU responsible for providing nonlinearity. Batch normalization and dropout were used as tricks to optimize model training. AP value was then the output of the last layer of the MLP decoder.

The training procedures of pentapeptides and decapeptides were similar, which were both single sequences and had a vocabulary of 20 amino acids. The length of the input sequence was fixed, and a sequence length of 5 and 10 was used to train pentapeptide and decapeptide, respectively. For training the Combo model, the input sequence length was fixed at 10, and the peptides of insufficient length were complemented by blank placeholders to fulfill the length. In the training of mixed pentapeptides, there was an extra word ‘+’ in the vocabulary to connect the two pentapeptides. Data augmentation was performed on the mixed pentapeptide data by swapping the positions of the two pentapeptides to eliminate the position effect.

Regarding the hyperparameters of models, the optimizer was set as stochastic gradient descent (SGD) and the learning rate as 0.2. Each model was trained for 200 epochs and batch size was set as 512. A validation set (a quarter of training data) was used to select the best‐performing model and test the performance of the models on a test set (the rest of the total data). The random seeds affected the initialization of the learnable parameters of the model as well as the partitioning of the dataset. To ensure the reliability of the experimental results, each experiment was repeatedly performed 10 times parallelly with different random seeds. The experimental results presented in this paper were the averages for each experiment.

For comparing the performance of deep‐learning and non‐deep‐learning models in predicting AP of peptides with enormous sequence space, five non‐deep‐learning models of support vector machine (SVM),^[^ ^32^ ^]^ random forest (RF),^[^ ^33^ ^]^ nearest neighbor (NN),^[^ ^34^ ^]^ Bayesian ridge (BR),^[^ ^35^ ^]^ and linear regression (LR) were also trained.^[^ ^36^ ^]^ The training of non‐deep learning models employed the open‐source package of ASCENDS.^[^ ^56^ ^]^

### Chemical Synthesis

Peptides were prepared by solid phase peptide synthesis (SPPS) using 2‐chlorotrityl chloride resin. The side chains of the corresponding N‐Fmoc protected amino acids were protected by different chemical groups. First, the C‐terminus of the first amino acid was conjugated to the resin. N, N’‐dimethyl formamide (DMF) containing 20% piperidine was used to remove Fmoc protected group. Next, O‐(Benzotriazol‐1‐yl)‐N, N, N’, N’‐tetramethyluronium hexafluorophosphate (HBTU) was used as a coupling reagent to couple the next amino acid to the free amino group. The growth of the peptide chain one by one followed the Fmoc SPPS protocol established above. After the last coupling step, excessive reagents were removed by a single DMF wash for 5 min, followed by five steps of washing using DCM for 1 min. The peptide was cleaved using 95% of trifluoroacetic acid (TFA) with 2.5% of triisopropylsilane and 2.5% of H_2_O for 45 min, with the resulting solution concentrated in vacuo. The residue was precipitated with ice‐cold diethyl ether and the resulting precipitate was centrifuged for 10 min at 4 °C at 5000 rpm min^−1^. Afterward, the supernatant was decanted, and the resulting solid was dissolved in H_2_O/CH_3_CN (1:1) for HPLC (High‐Performance Liquid Chromatography) separation.

HPLC separation was conducted at Agilent 1260 Infinity II Manual Preparative Liquid Chromatography system using a C18 RP column with CH_3_CN (0.1% of trifluoroacetic acid) and water (0.1% of trifluoroacetic acid) as the eluents. Flow: 10.0 mL min^−1^. Column temperature: 37 °C. Eluant: A‐water (0.1% TFA), B‐CH_3_CN (0.1% TFA). Method: 0–11 min, 5−80% B, 11–13 min, 80−100% B, 13–16 min, 100% B, 16–18 min, 100−5% B. LC‐MS was conducted at the Agilent InfinityLab LC/MSD system.

### TEM Characterization

The negative staining technique was employed to observe the morphologies formed by peptides, with TEM samples prepared at 25 °C. A micropipette was used to load 10 µL of sample solution to a carbon‐coated copper grid. The excess solution was removed by a piece of filter paper. After rinsing the grid with the deionized water, uranyl acetate was used to stain the sample for 1 min and then the grid was rinsed with deionized water again. The samples were dried overnight in a desiccator and then conducted on a Talos L120C system, operating at 120 kV.

## Conflict of Interest

The authors declare no conflict of interest.

## Author Contributions

W.L., S.Z.L., and H.W. conceived the initial idea and supervised coarse‐grained molecular dynamics simulation, machine learning, and experimental work, respectively. J.W. and S.Z. performed the coarse‐grained molecular dynamics simulations, analyzed the data, and wrote the manuscript. W.L. revised the manuscript and provided insights on discussions. Z.L. performed the AI model training and prediction and wrote the manuscript regarding the methods of model training. T.X. performed the chemical synthesis of peptides, characterized with TEM, and wrote the manuscript regarding the methods of experiments. J.W., Z.L., S.Z., and T.X. contributed equally to this work.

## Supporting information

Supporting Information

Supporting Information

## Data Availability

The data that support the findings of this study are available from the corresponding author upon reasonable request.
