# Supplementary material for: Deep Learning Empowers the Discovery of Self‐Assembling Peptides with Over 10 Trillion Sequences
Source: Adv Sci (Weinh). 2023 Sep 25;10(31):2301544. doi: 10.1002/advs.202301544 (PMC10625107; doi:10.1002/advs.202301544)
Supplement: Supplementary file 1 — Supporting Information [file ADVS-10-2301544-s001.pdf]

Supporting Information

Deep Learning Empowers the Discovery of Self-Assembling Peptides with over Ten Trillion Sequences

Jiaqi Wang[#], Zihan Liu[#], Shuang Zhao[#], Tengyan Xu[#], Huaimin Wang, Stan Z. Li[3]*, Wenbin Li**

[#]Co-first author
*Co-corresponding author

Jiaqi Wang, Shuang Zhao, Wenbin Li, Research Center for Industries of the Future, Westlake University, Hangzhou 310030, China; School of Engineering, Westlake University, Hangzhou 310030, China
Email: liwenbin@westlake.edu.cn

Zihan Liu, Stan Z. Li, AI Lab, Research Center for Industries of the Future, Westlake University, Hangzhou 310030, China

Tengyan Xu, Huaimin Wang, Department of Chemistry, School of Science, Westlake University, Hangzhou 310030, China; Institute of Natural Sciences, Westlake Institute for Advanced Study, 18 Shilongshan Road, Hangzhou 310024, Zhejiang Province, China.

Content

Supplementary Discussions (SD) 3

SD1: Evaluation of secondary structure and simulation duration 3

SD2. Improvement of score function AP_H (to AP_{HC}) 7

Supplementary Figures for Main Body 12

Supplementary Tables for Main Body 23

Supplementary Discussions (SD)

SD1: Evaluation of secondary structure and simulation duration

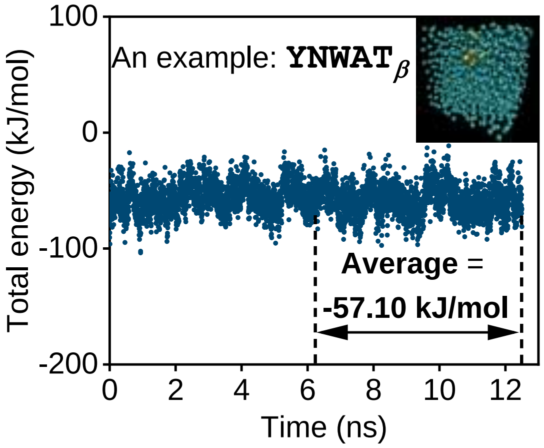

Figure SD1. Evaluation of total energy of a single pentapeptide/decapeptide with either α or β conformation in water at 300 K (example: YNWAT with β-strand conformation).

Both pentapeptides and decapeptides with α-helix conformation have lower total energy in simulated conditions (Figure SD2a, b). Although most peptides with α-helix conformation carry higher bonded energies (Figure SD2c, d), the non-bonded interactions (Figure SD2e, f) dominate the self-assembling and final morphology of peptides in solvent[5]. Therefore, α-helix should be a reasonable starting conformation in CGMD simulations (Figure 1a).

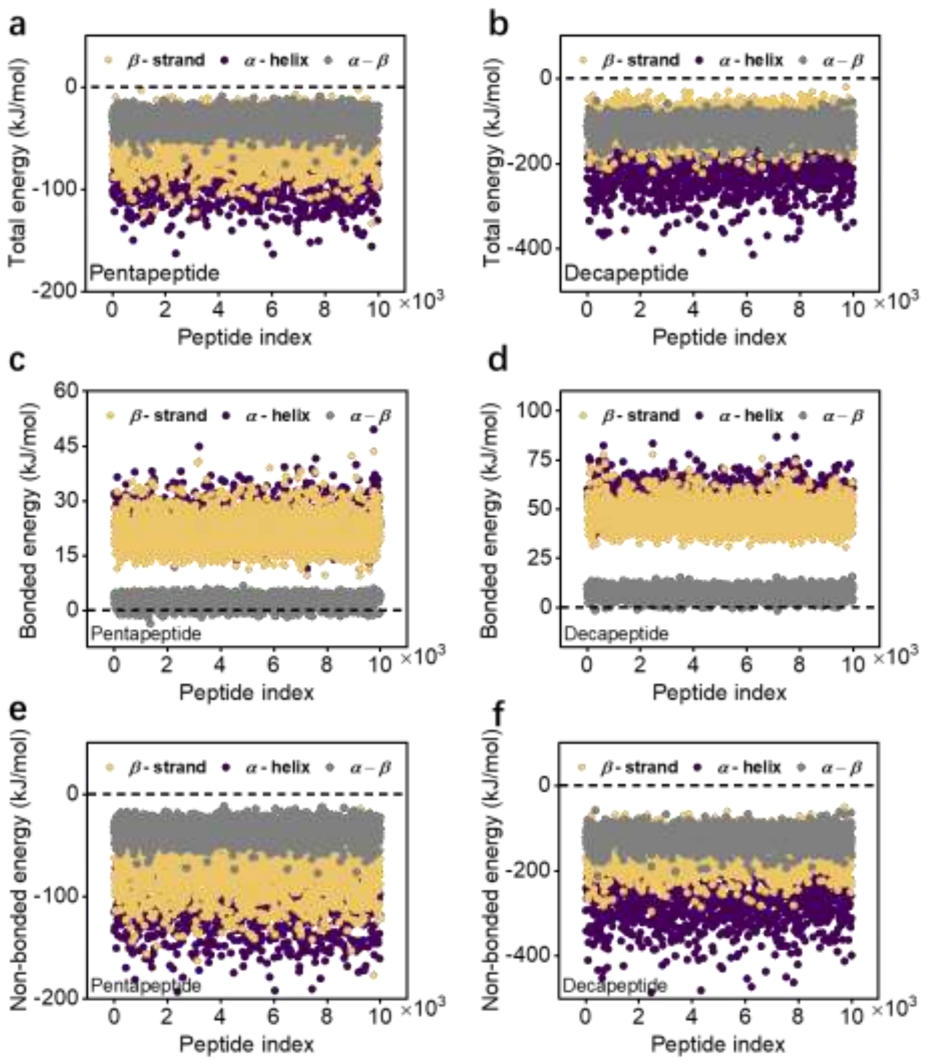

Figure SD2. a-b. Total energy of a single pentapeptide/decapeptide with either *α* or *β* conformation in water at 300 K. Both pentapeptides and decapeptides with *α*-helix exhibit lower total energy. **c-d.** Bonded energy. Most pentapeptide and decapeptides have higher bonded energy with *α*-helix conformation. **e-f.** Non-bonded energy. All pentapeptides and decapeptides have lower non-bonded energy with *α*-helix conformation in water at 300 K, suggesting that *α*-helix is a reasonable starting conformation in aggregation simulation.

To further identify the effect of conformation on aggregation, we perform simulations on randomly selected 620 pentapeptides with both *β*-strand and *α*-helix conformations for 125 ns. The mean absolute difference of 620 AP values between the two conformations is 0.08 (Figure SD3a), evidencing that most pentapeptides with different conformations generate similar AP.

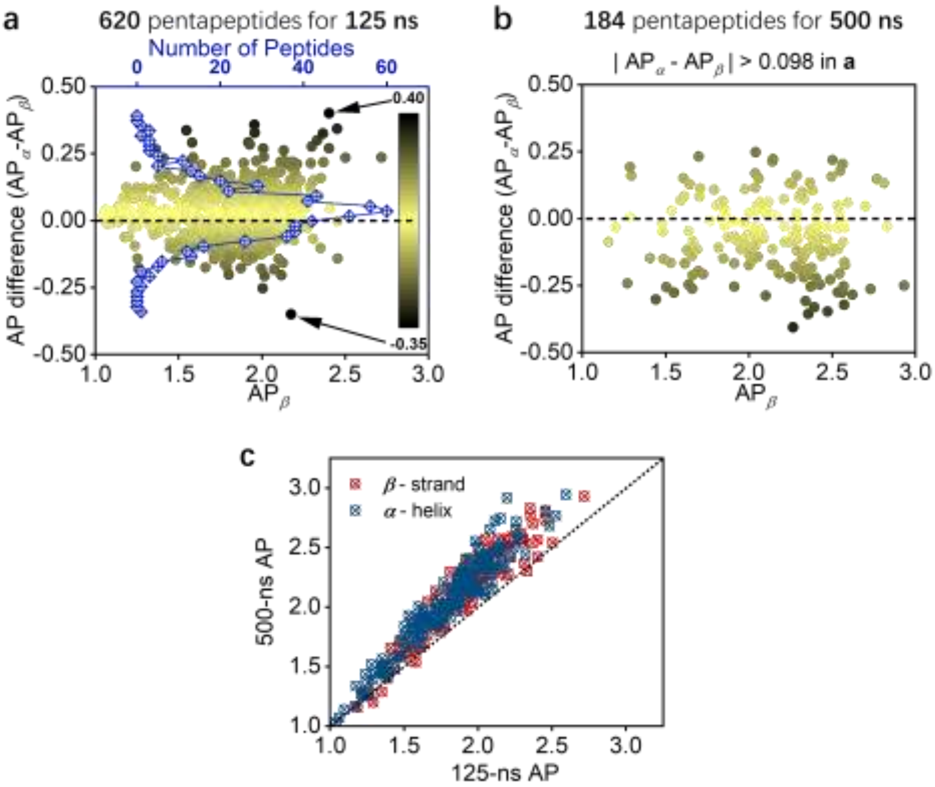

Figure SD3. a. AP difference (AP_α-AP_β, filled circles) between β-strand and α-helix conformation and number distribution (blue-edged diamonds) of 620 pentapeptides within each AP difference bin, calculated at 125 ns. A maximum of 0.40, a minimum of -0.35, and a mean absolute difference of 0.08 is achieved. The AP difference is divided into 40 bins with a bin size of 0.01878, and 70% of peptides are found to have an absolute AP difference of 0.10, 86% are within the absolute difference of 0.15. **b.** AP difference of 184 peptides (selected from **a** with .AP_α-AP_β. > 0.098) calculated at 500 ns. A mean absolute difference of 0.12 is achieved. **c.** AP of both β-strand and α-helix conformation calculated at 125 and 500 ns. APs' remain relatively constant, with 500-ns AP just slightly larger than 125-ns AP due to longer simulation time promoting further aggregation. It is thus reasonable to choose 125 ns as simulation duration in the initial phase of generating training data, as a good compromise between computational cost and simulation accuracy.

To eliminate the non-convergence effect on AP difference due to simulation time, we pick 184 pentapeptides with AP difference between different conformations larger than 0.098 at 125 ns, then perform aggregation simulations for another 375 ns to a total of 500 ns. The mean absolute difference between the two conformations of 184 pentapeptides is 0.12 at 500 ns (Figure SD3b). We thus conclude that setting different conformations has negligible effect on the AP of short peptides calculated by CGMD simulations. Comparing 125-ns and 500-ns

AP of the 184 peptides, they remain relatively constant with 500-ns AP slightly larger than 125-ns AP (Figure SD3c), but no peptides are observed to change from "no aggregating" to "strongly aggregating" or *vice versa*, therefore it is appropriate to choose 125 ns for generating training data, as a reasonable compromise between computational cost and simulation accuracy.

In addition to initial energy of a single peptide in water solvent, energy of peptide with two conformations after aggregation is also examined, as an alternative approach to investigate possible secondary structure transition, which is calculated by averaging the energies over the last 150 ns duration (from 350 ns to 500 ns, Figure SD4a). It is found that 59 peptides out of the total 184 peptides with β-strand conformation have lower total energy than that of α-helix conformation after aggregation (Figure SD4b), although all peptides have higher initial energy with β-strand conformation. This could indicate a transition of secondary structure from α-helix to β-sheet or the formation of intermolecular β-sheet conformation during self-assembling process[6]. Investigation of secondary structure transition/formation during self-assembly falls out of scope of this research and will be conducted in future work.

Examining the difference of Lennard-Jones potential with respect to AP difference (Figure SD4c), a clear linear relation can be observed as marked by red line, indicating that from the energy perspective, part of peptides with β-sheet conformation could bear stronger propensity for aggregation. The inset of Supplementary Figure SD4c illustrates no discernable discrepancy between Lennard-Jones potential difference and total energy difference, demonstrating that the non-bonded interactions of Lennard-Jones potential dominates the self-assembly process of studied pentapeptides, which coincides well with available literature[5]. It should be acknowledged that self-assembly of peptides can be driven synergistically by various interactions, such as hydrogen bonding, π-π stacking, hydrophobicity, Coulombic interaction, *etc*., and this Lennard-Jones interaction can be different combinations of these interactions (exclusive of Coulombic interaction). Regarding peptides with charged amino acids, the Coulombic interaction is found more than one order of magnitude smaller than the Lennard-Jones potential, and the bonded interaction (bond stretching, bond angle, proper dihedrals, and improper dihedrals interactions) is generally one order of magnitude smaller than the non-bonded interactions (Lennard-Jones and Coulombic) for all peptides.

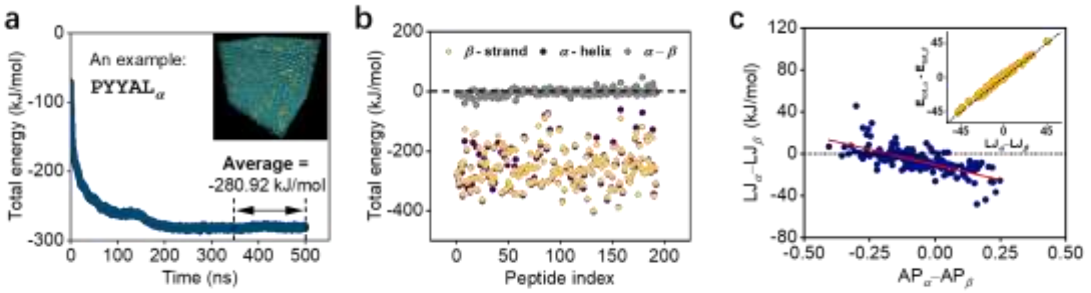

Figure SD4. a. Energy evolution during the self-assembling process of 150 individual PYYAL$_\alpha$ (α-helix conformation) peptides in water for 500 ns. **b.** Final energy of 184 pentapeptides after aggregation, averaged by both the number of peptides in aggregation simulation (*i.e.*, 150) and simulation duration from 350 ns to 500 ns. 59 pentapeptides with β-strand conformation has lower energy after aggregation, indicating possible transitions of secondary structure during self-assembly process. **c.** Lennard-Jones potential energy difference with respect to AP difference, with the inset showing total energy difference versus Lennard-Jones potential energy difference. The Lennard-Jones potential dominates the self-assembly process and part of peptides exhibit stronger aggregation propensity with β-strand conformation.

SD2. Improvement of score function AP_H (to AP_{HC})

Since AP values generated by CGMD cannot provide sufficient details for distinguishing between aggregation, precipitation, crystallization, or self-assembly to ordered structure, a hydrophilicity-corrected score AP_H[7] has been developed as $AP_H = AP'^{\alpha} \times (\log P)'^{\beta}$ for selecting appropriate peptides for more practical use. The prime symbol denotes normalization between 0 to 1 and logP is hydrophilicity calculated directly from the Wimley-White whole-residue scale[8]. We list the rank of $AP_H^{2\text{-}1}$ ($\alpha = 2, \beta = 1$) and $AP_H^{2\text{-}0.5}$ ($\alpha = 2, \beta = 0.5$) scores in Table SD1, calculated based on the original score function and the AP_{prd} obtained in this work. The ranking of $AP_H^{2\text{-}1}$ and $AP_H^{2\text{-}0.5}$ of non-aggregated peptides are significantly overestimated by the original score functions, especially for KKFDD, EPYYK, and KDPYY with both high logP' and AP'. Examining the available experimental data[9], we found that aggregated peptides normally have relatively low logP' values (0.2845 < logP' < 0.5410, with a mean of 0.4113) and while non-aggregated peptides have relatively high logP' values (0.3449 < logP' < 0.7557, with a mean of 0.5346) (Figure SD5a). The original score function then assigns high scores to peptides with both high AP' and logP', leading to overestimation of scores of soluble peptides. It can be deduced that a high logP' indicates high solubility and the peptides will thus possibly

not aggregate in real world, while the CGMD simulation with Martini force field is incapable of describing intermolecular interactions with full precision and could generate high AP scores for peptides with high logP', inducing bias on selecting peptides with high solubility. However, it should be noted that peptide self-assembling can be promoted by various interactions other than hydrophobicity, such as hydrogen bonding, π-π stacking, electrostatic interactions, *etc.*, thus, it is possible that a high logP' incurs relatively high AP values (such as KFAFD and VKVEV). It is also worth being noted that for aggregated peptides in experiment, the ratio of AP_{prd}'/logP' is 1.4085, while for non-aggregated peptides, the ratio is 1.0980, which is quite reasonable since with a relatively small logP' (*i.e.*, neither soluble nor insoluble) and relatively large AP (*i.e.*, medium aggregation without precipitation), the peptides have the highest possibility of self-assembling. The ratio can be a reference value in selecting self-assembling peptides and has been listed in Table SD1 as well.

To correct the bias of the original score functions to highly soluble peptides, we introduce a penalty factor for hydrophilicity, *i.e.*, $e^{-\frac{(\mathrm{logP}'-\mu)^2}{2\sigma^2}}$, to the original score function:

$$\mathrm{AP_{HC}} = \mathrm{AP}'^{\alpha} \times (\mathrm{logP}')^{\beta} \times e^{-\frac{(\mathrm{logP}'-\mu)^2}{2\sigma^2}} \qquad (1)$$

Here, α = 2 and β = 0.5. μ (= 0.4113) and σ^2 (= 0.0657) are the average and variance of logP' of aggregated peptides among the 26 pentapeptides aforementioned, respectively.

In the selection of aggregated peptides or the exclusion of non-aggregated peptides, we want the rankings (the score is ranked in descending order, with the highest score having a rank of 1, while the lowest score having a rank of the last) of the scores of possibly aggregated peptides to increase while those of non-aggregated peptides to decrease as much as possible. Comparing the original and corrected AP_H (Figure SD5b, c), introducing the penalty factor improves the ranking of most aggregated peptides, while lowering that of non-aggregated peptides, leading to more accurate selection. Although the rankings of aggregated pentapeptides VVVVV, VKVFF, VKVEV and non-aggregated pentapeptides VKVKV, WKPYY, PTPCY, and PPPHY (indicated by symbol ⊗ in Figure SD5b, c) have been lowered due to the large deviation of logP' from the mean value, the ranking is still close to the original one. Care thus should be taken that penalty imposed here may degrade the ranking of peptides with relatively high AP values containing V, P, and Y amino acids with logP' values deviating from mean value. In general, the penalty factor improves the performance of the original score function (19 over 26 peptides) by decreasing the weight of logP in the more

hydrophilic range. There is no doubt that the score function can be further improved by either optimizing the weighting factor or including more parameters denoting other interactions or the formation of β-sheet structures or ordered morphologies after aggregation. For example, Rohit Batra has tentatively included a β-sheet propensity parameter (reported by Chou and Fasman[10]) with an associated weight in the score function and found the performance of score function is improved, but selection of associated weight of β-sheet propensity is still empirical and undesired bias can be introduced[9]. Another possible approach is to select a mass of peptides with great diversity based on the current score function, and experimentally synthesize them for examination of aggregation or not, and subsequently train AI models bearing the underlying physical laws of aggregation for prediction. With increasing amount of data, the performance of AI models could be iteratively improved and capable of suggesting peptide sequences with greater aggregation propensity or predicting the aggregation propensity of interested peptides.

Table SD1. Aggregation-related information of 26 experimentally tested pentapeptides. Agg. = 1 (0) denotes aggregation (non-aggregation) in experiment; logP' is rescaled hydrophilicity between 0 to 1; AP_{prd}, AP_{sim}, AP_{prd}', and AP_{rep} are predicted AP, simulated AP, rescaled predicted AP, and reported AP values in reference 9, respectively; Ratio is calculated by AP_{prd}'/logP', which can assist in selecting aggregating peptides; r-AP_H^{2-1} and r-$AP_H^{2-0.5}$ are the ranking of AP_H value calculated by original score functions with parameters of $\alpha = 2$, $\beta = 1$ and $\alpha = 2$, $\beta = 0.5$, while r-AP_{HC} is the ranking of AP_{HC} score calculated by the revised score function with parameters in Equation 1, and the percentage is the improvement of r-AP_{HC} compared to r-$AP_H^{2-0.5}$. For aggregated peptides, it is calculated by (r-$AP_H^{2-0.5}$ – r-AP_{HC})/r-$AP_H^{2-0.5}$, while for non-aggregated peptides, it is calculated by (r-AP_{HC} – r-$AP_H^{2-0.5}$)/r-$AP_H^{2-0.5}$, the minus sign denotes performance degradation of r-AP_{HC} compared with r-$AP_H^{2-0.5}$.

Index	Pep	Agg.	logP'	AP_{prd}	AP_{sim}	AP_{prd}'	AP_{rep}[9]	Ratio	r-AP_H^{2-1}	r-$AP_H^{2-0.5}$	r-AP_{HC}	Percentage
1	**FKIDF**	**1**	0.4311	2.10	2.03	0.7767	1.9000	1.8017	29269	15740	6464	58.93%
2	**FFEKF**	**1**	0.4101	2.13	2.15	0.7937	2.2400	1.9353	32098	10537	4032	61.73%
3	**KWEFY**	**1**	0.4318	2.15	2.19	0.8044	1.9200	1.8628	8790	2823	1100	61.03%
4	**SYCGY**	**1**	0.3707	2.06	2.23	0.7518	2.4200	2.0280	242198	177167	82329	53.53%
5	**RWLDY**	**1**	0.4136	2.07	2.19	0.7590	2.1100	1.8352	83168	57945	21552	62.81%
6	**KWMDF**	**1**	0.4335	2.12	2.20	0.7851	1.9700	1.8110	19410	9172	3843	58.10%
7	**KFFFE**	**1**	0.4101	2.19	2.23	0.8304	1.9500	2.0248	6870	871	259	70.26%
8	**FKFEF**	**1**	0.4101	2.19	2.16	0.8340	2.1200	2.0337	5719	659	174	73.60%
9	**VVVVV**	**1**	0.2845	2.05	2.16	0.7405	2.0600	2.6027	1331273	775313	939506	-21.18%
10	**VKVFF**	**1**	0.3110	2.17	2.13	0.8198	1.9500	2.6361	246409	47215	60410	-27.95%
11	**KFAFD**	**1**	0.4876	2.01	1.95	0.7166	1.8600	1.4697	48219	98664	72019	27.01%
12	**VKVEV**	**1**	0.5410	1.82	1.76	0.5966	1.8400	1.1028	444518	1250400	1409118	-12.69%
13	VKVKV	0	0.5120	1.45	1.50	0.3588	1.3500	0.7008	2711292	2713741	2673612	-1.48%
14	KVKVK	0	0.6258	1.21	1.19	0.2047	1.1900	0.3271	3005905	3026020	3049571	0.78%
15	KKFDD	0	0.7546	1.95	1.92	0.6775	1.6400	0.8979	14	13589	2165662	15836.88%
16	DPDPD	0	0.7557	1.00	1.00	0.0696	1.0100	0.0920	3192981	3193684	3195219	0.05%
17	RVSVD	0	0.5389	1.86	1.87	0.6219	1.6600	1.1540	251543	907341	1075673	18.55%
18	WKPYY	0	0.3449	2.20	2.22	0.8371	2.1100	2.4271	72175	6591	5464	-17.10%
19	PTPCY	0	0.3578	2.13	2.10	0.7972	2.5400	2.2280	127695	36317	20232	-44.29%
20	PPPHY	0	0.3585	2.22	2.20	0.8510	2.4600	2.3737	30065	1571	1049	-33.23%
21	KDHFY	0	0.5089	2.09	2.20	0.7711	2.3000	1.5153	1457	3010	6599	119.24%
22	YTEYK	0	0.5483	1.96	1.98	0.6858	2.1300	1.2508	32892	150734	325201	115.74%
23	EPYYK	0	0.5445	2.06	2.02	0.7489	2.2600	1.3755	1144	5680	32088	464.93%
24	YDPKY	0	0.5449	2.01	2.07	0.7187	2.2400	1.3190	7557	34192	109241	219.49%
25	KDPYY	0	0.5449	2.09	2.11	0.7703	2.2200	1.4137	209	1132	12210	978.62%
26	YEPYK	0	0.5445	1.99	2.03	0.7020	2.1600	1.2892	18099	79326	192655	142.86%

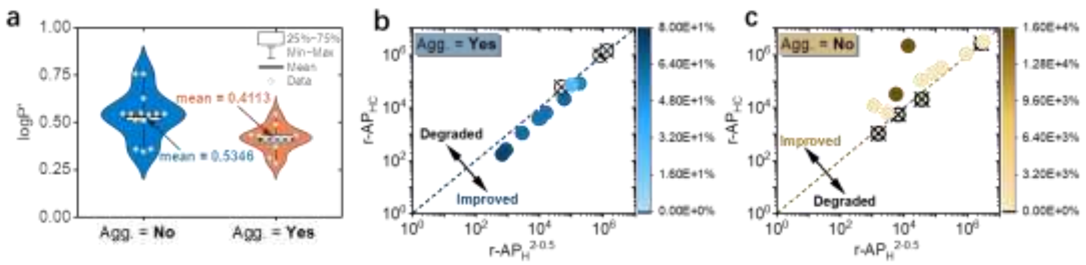

Figure SD5. a. Distribution of logP' of aggregated and non-aggregated peptides. A mean value of 0.4113 and 0.5346 is obtained for aggregated and non-aggregated peptides. **b, c.** Comparison of original and corrected AP_H ($AP_H^{2\text{-}0.5}$ vs AP_{HC}) score for aggregated and non-aggregated peptides, respectively. AP_{HC} score exhibits performance improvement over 19 peptides out of the total 26. The color bar represents the performance improvement, calculated by (r-$AP_H^{2\text{-}0.5}$ – r-AP_{HC})/r-$AP_H^{2\text{-}0.5}$ for aggregated peptides, and by (r-AP_{HC} – r-$AP_H^{2\text{-}0.5}$)/r-$AP_H^{2\text{-}0.5}$ for non-aggregated peptides.

Supplementary Figures for Main Body

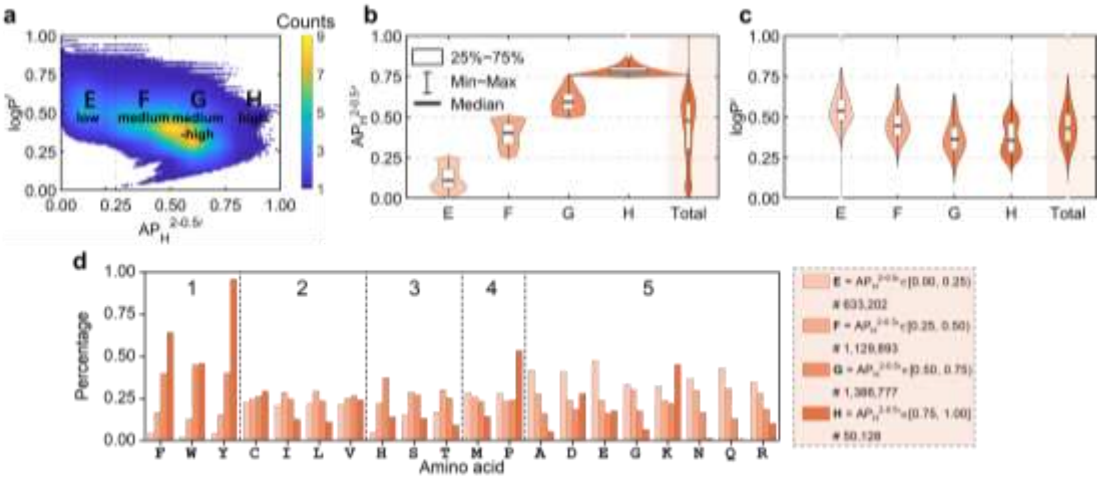

Figure S1. a. Relation between $AP_H^{2\text{-}0.5\prime}$ and logP'. Four ranges of $AP_H^{2\text{-}0.5\prime}$ is indicated by low-E, medium-F, medium-high-G, and high-H. The number of peptides in each range is 633202, 1129893, 1386777, 50128. **b.** Distribution of $AP_H^{2\text{-}0.5\prime}$ within four $AP_H^{2\text{-}0.5\prime}$ ranges. **c.** Distribution of logP' within four $AP_H^{2\text{-}0.5\prime}$ ranges. **d.** Distribution of 20 amino acids in within four $\mathbf{AP_H^{2\text{-}0.5\prime}}$ ranges (E, F, G, and H). The amino acids have been divided into five groups according to their contribution to aggregation, in terms of $\mathbf{AP_{prd}}$'.

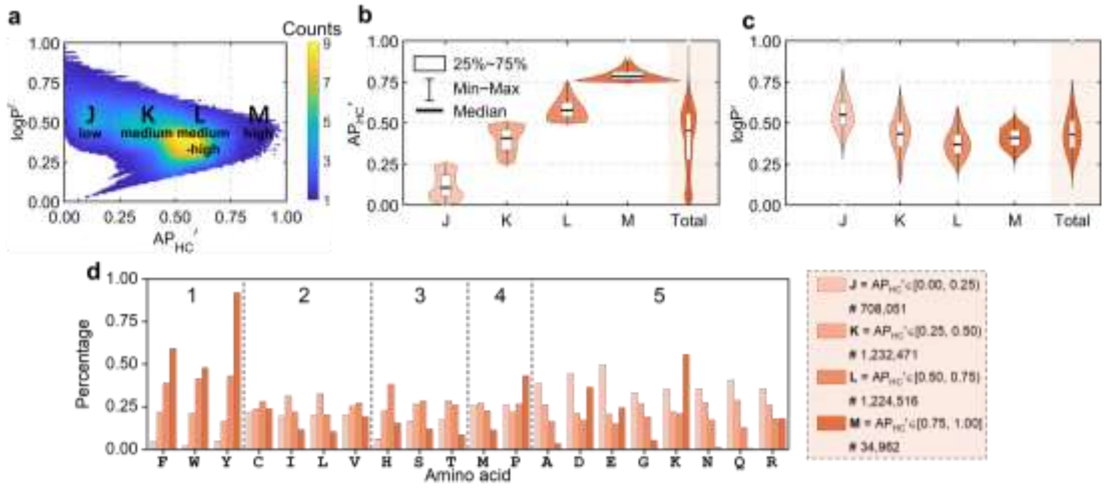

Figure S2. a. Relation between AP_{HC}' and logP'. Four ranges of AP_{HC}' is indicated by J, K, L, and M. The number of peptides in each range is 708051, 1232471, 1224516, 34962. **b.** Distribution of AP_{HC}' within four AP_{HC}' ranges. **c.** Distribution of logP' within four AP_{HC}' ranges. **d.** Distribution of 20 amino acids within four **AP_{HC}'** ranges (J, K, L, and M). The amino acids have been divided into five groups according to their contribution to aggregation, in terms of **AP_{prd}'**.

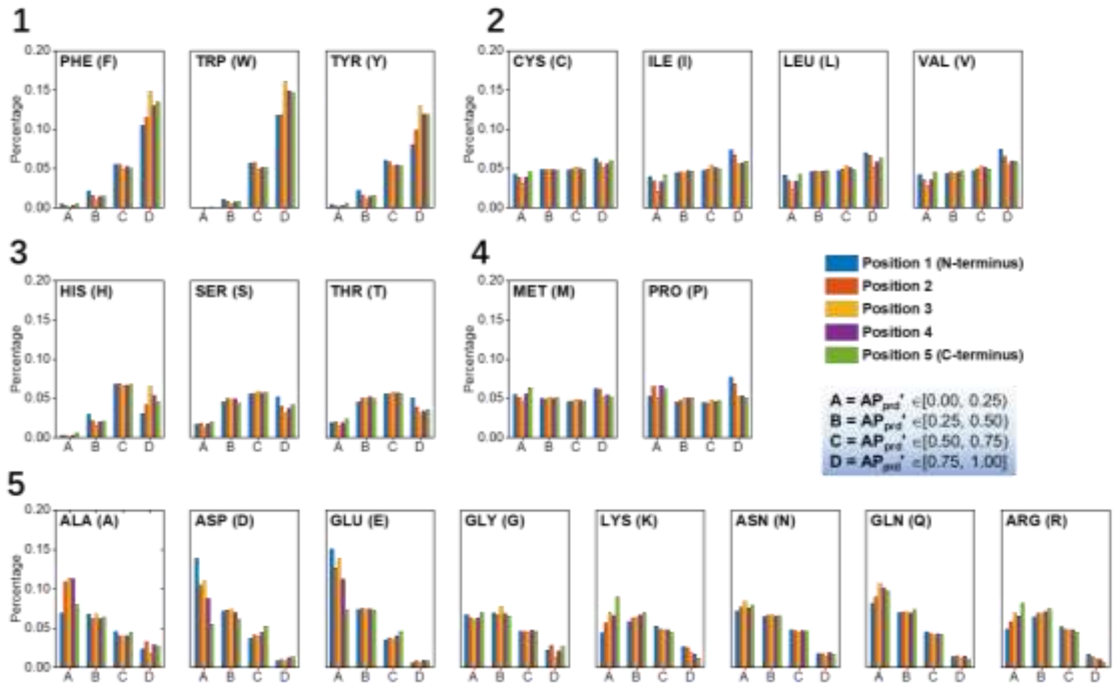

Figure S3. Distribution of each amino acid at each position, calculated based on **AP$_{prd}$'**.

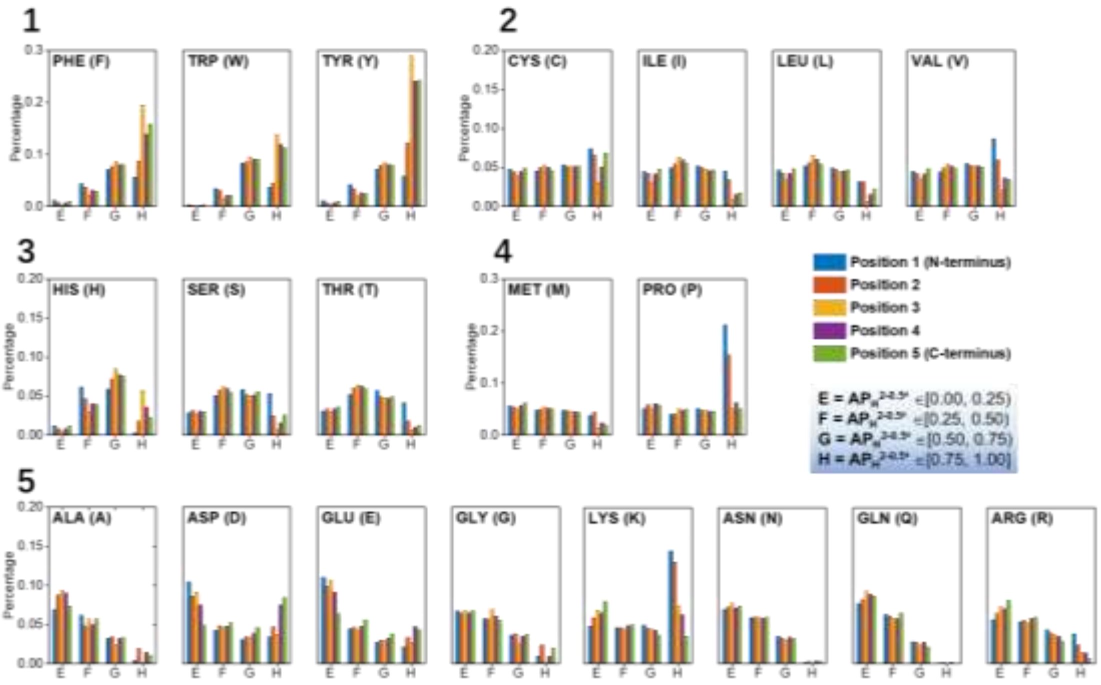

Figure S4. Distribution of each amino acid at each position, calculated based on $\mathbf{AP_H^{2\text{-}0.5'}}$.

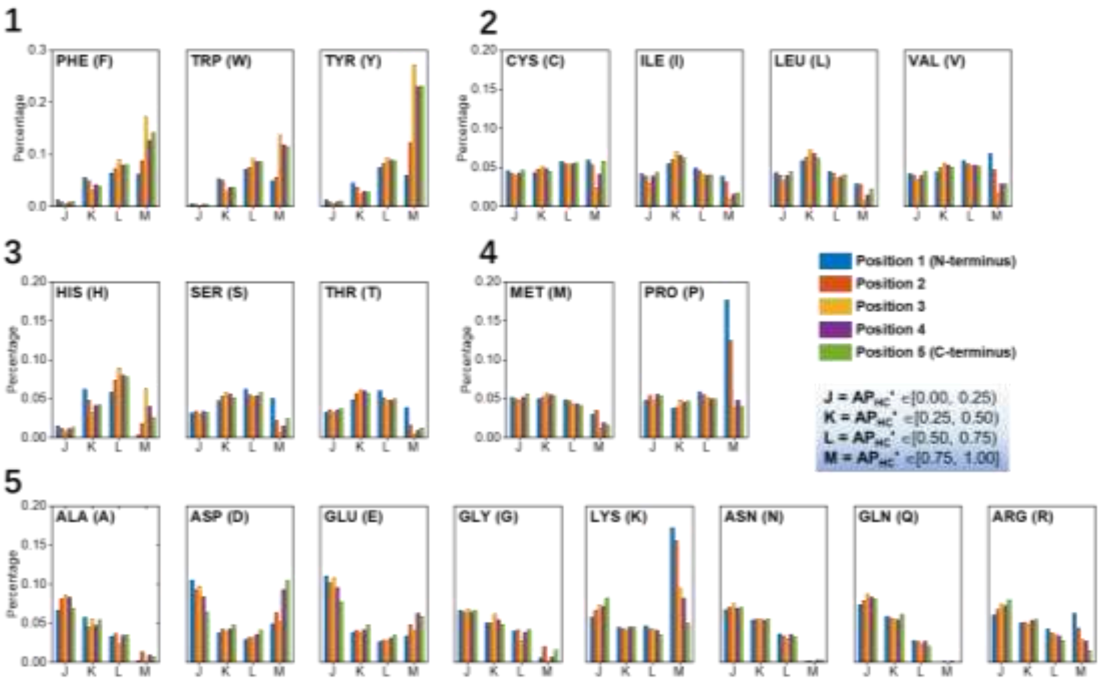

Figure S5. Distribution of each amino acid at each position, calculated based on $\mathbf{AP_{HC}'}$.

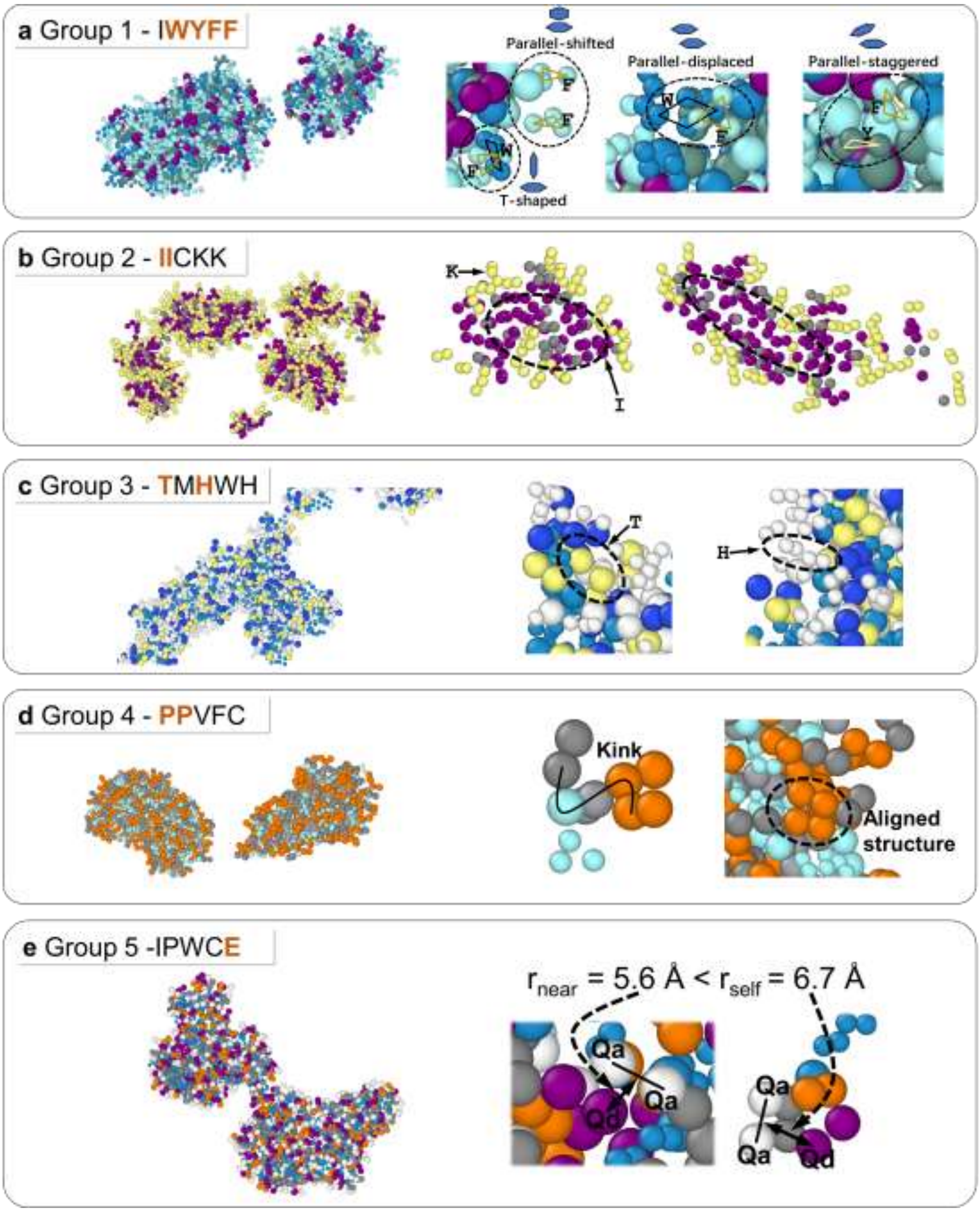

Figure S6. Examples illustrating five different mechanisms promoting aggregation: **a.** π-stacking. **b.** hydrophobicity. **c.** polarity. **d.** kink conformation of Proline. **e.** Columbic interaction.

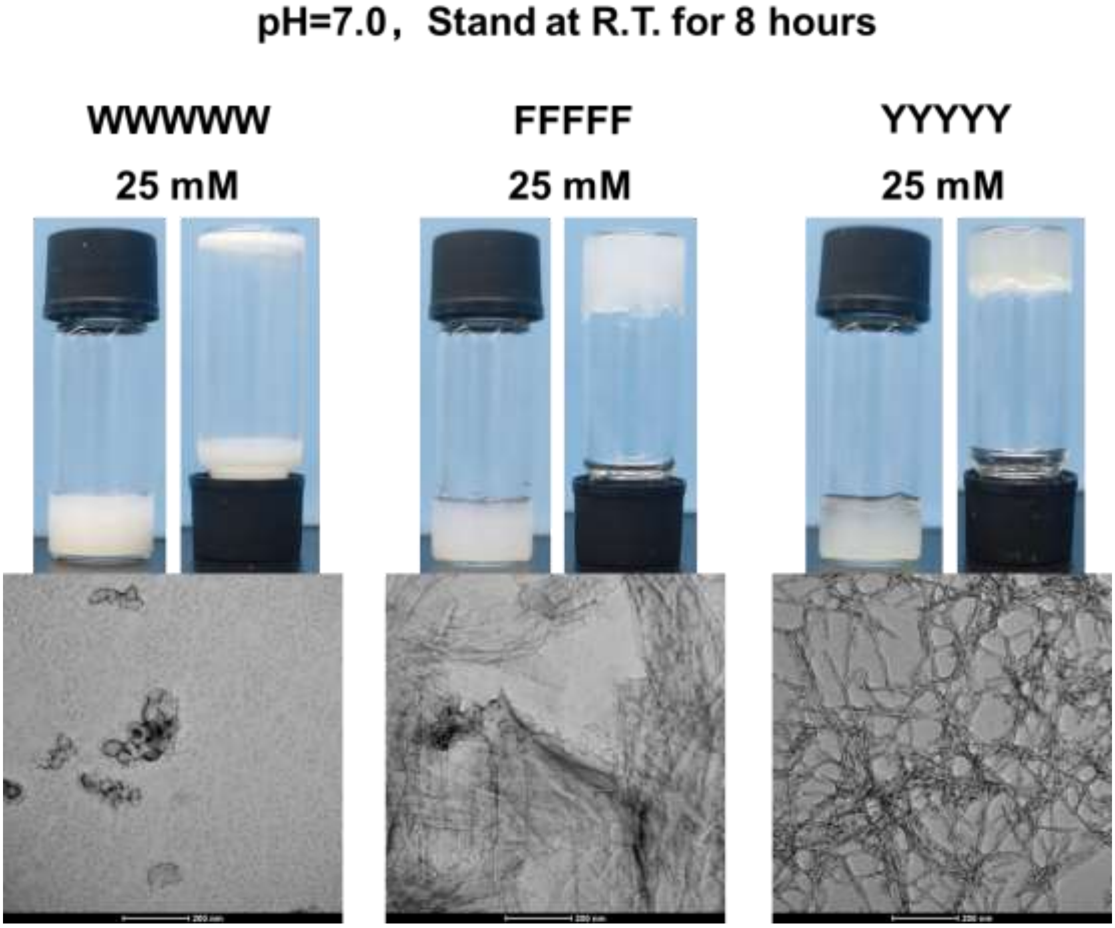

Figure S7. Photographs and TEM images of WWWWW, FFFFF, and YYYYY peptides in water. WWWWW forms suspensions with amorphous precipitates while FFFFF and YYYYY form hydrogels with nanosheet and nanofiber structures, respectively. All results are obtained at pH = 7 after 8 hours of standing at room temperature (R.T.).

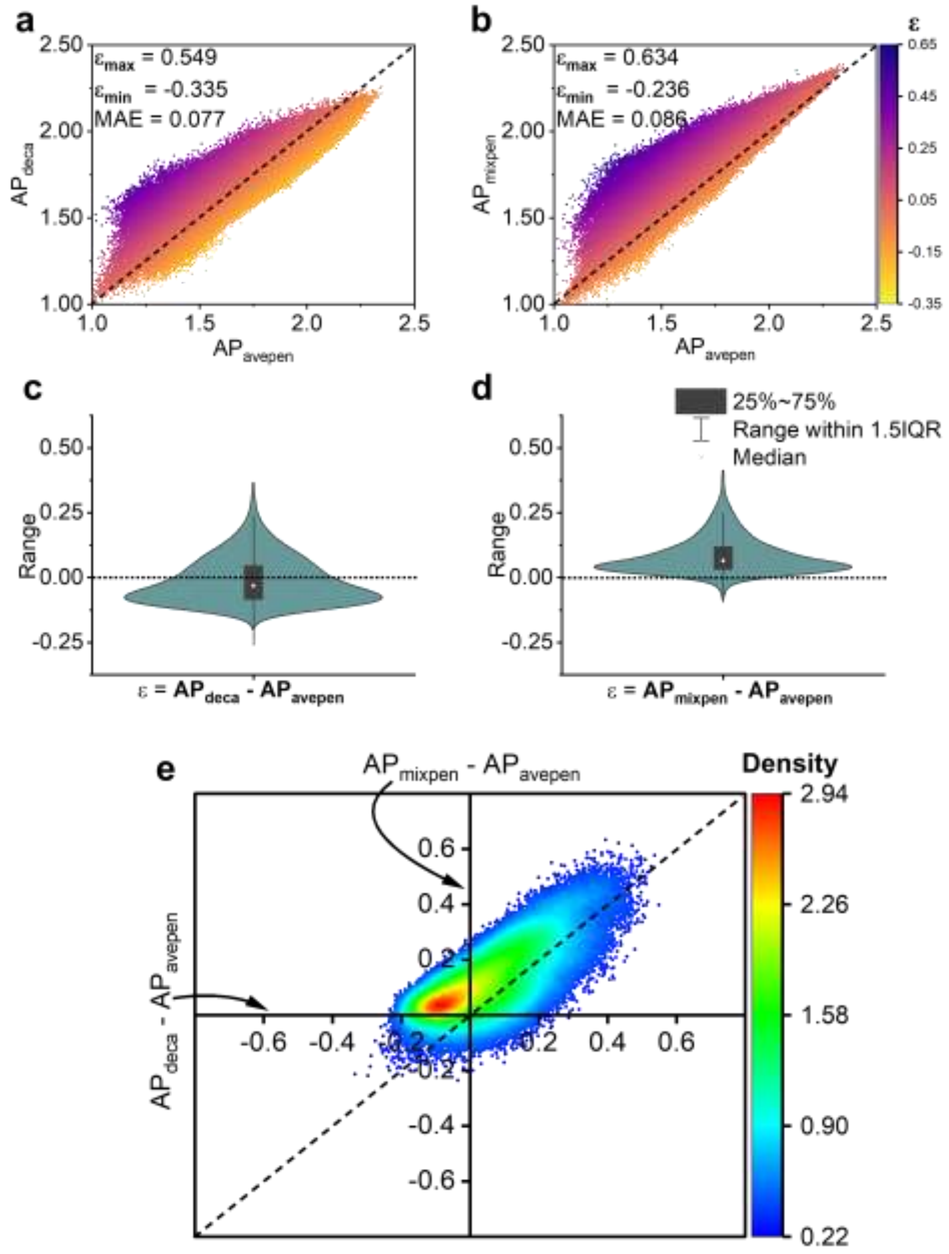

Figure S8. a. Transferability relation between AP of decapeptides (AP_{deca}) and averaged AP of two pentapeptides [$AP_{avepen} = (AP_{penta1} + AP_{penta2})/2$]**. b.** Transferability relation between AP of mixed pentapeptides (AP_{mixpen}) and AP_{avepen}. **c-d.** Distribution of AP difference, *i.e.*, AP_{deca} - AP_{avepen} and AP_{mixpen} - AP_{avepen}. **e.** Relation between AP_{deca} - AP_{avepen} and AP_{mixpen} - AP_{avepen}. Data of AP_{deca} and AP_{penta} employed here are predicted by the combo model and data of AP_{mixpen} is predicted by the mixed model. Totally 1 million groups of data of AP_{deca}, AP_{mixpen}, and AP_{avepen} are studied.

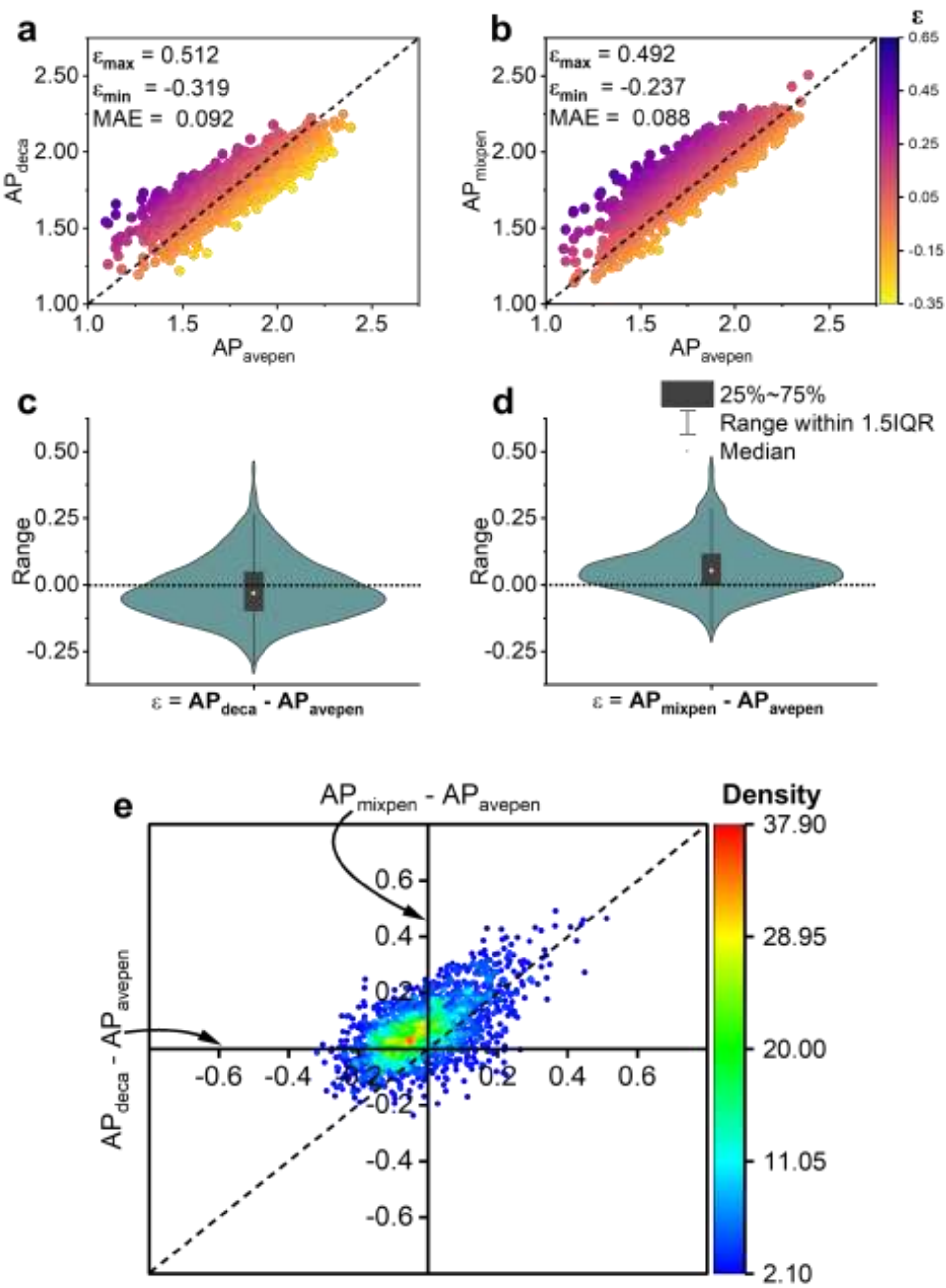

Figure S9. a. Transferability relation between AP of decapeptides (AP_{deca}) and averaged AP of two pentapeptides [$AP_{avepen} = (AP_{penta1} + AP_{penta2})/2$]**. b.** Transferability relation between AP of mixed pentapeptides (AP_{mixpen}) and AP_{avepen}. **c-d.** Distribution of AP difference, *i.e.*, AP_{deca} - AP_{avepen} and AP_{mixpen} - AP_{avepen}. **e.** Relation between AP_{deca} - AP_{avepen} and AP_{mixpen} - AP_{avepen}. Data of AP_{deca} and AP_{penta} employed here are generated by coarse-grained molecular dynamics. Totally 2253 groups of data of AP_{deca}, AP_{mixpen}, and AP_{avepen} are studied, for comparing the results of predicted data in Supplementary Figure 8.

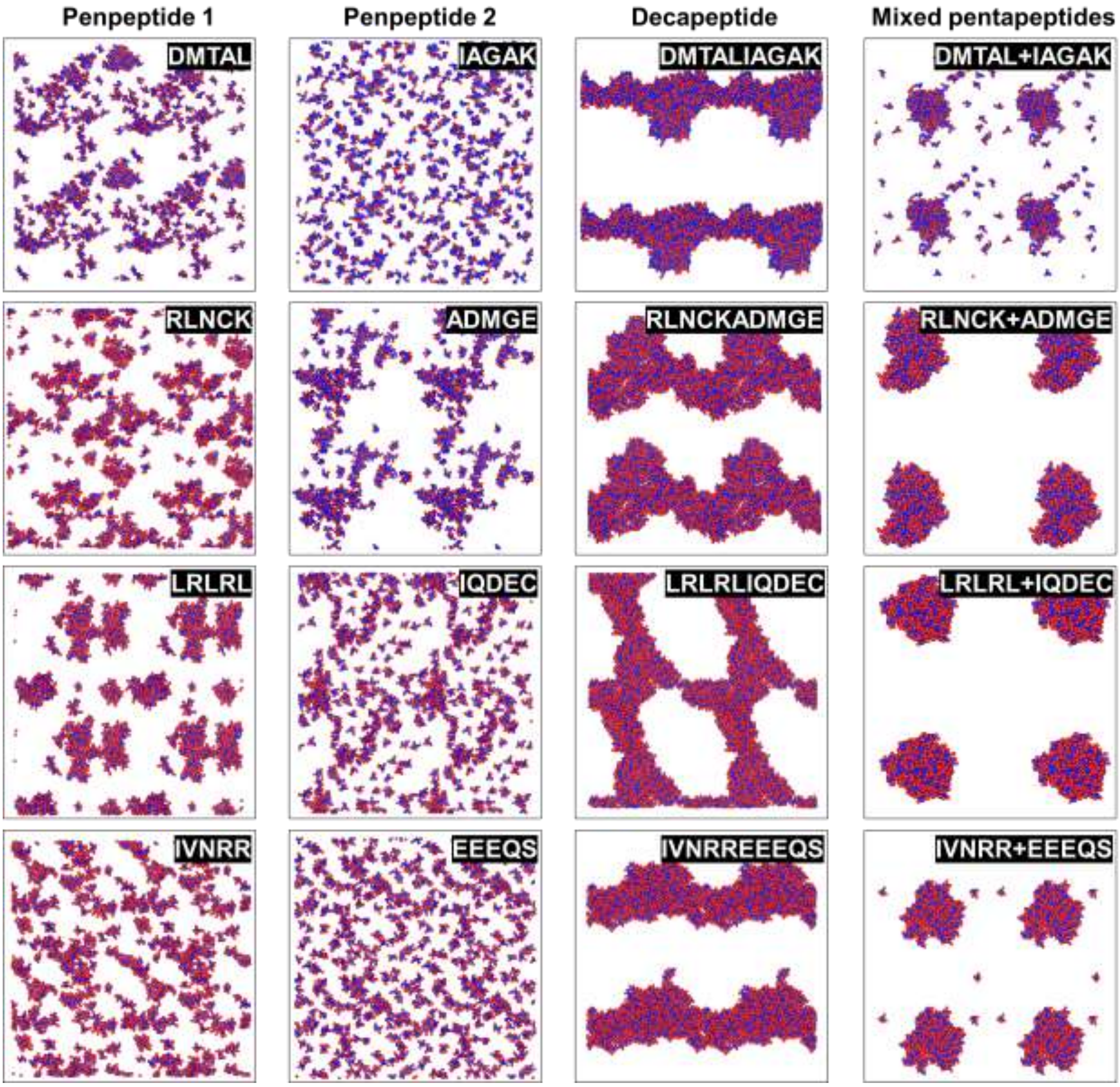

Figure S10. Computational morphologies of four groups of pentapeptides (simulated for 1.25 μs), decapeptides (simulated for 6.25 μs) and mixed pentapeptides (simulated for 1.25 μs).

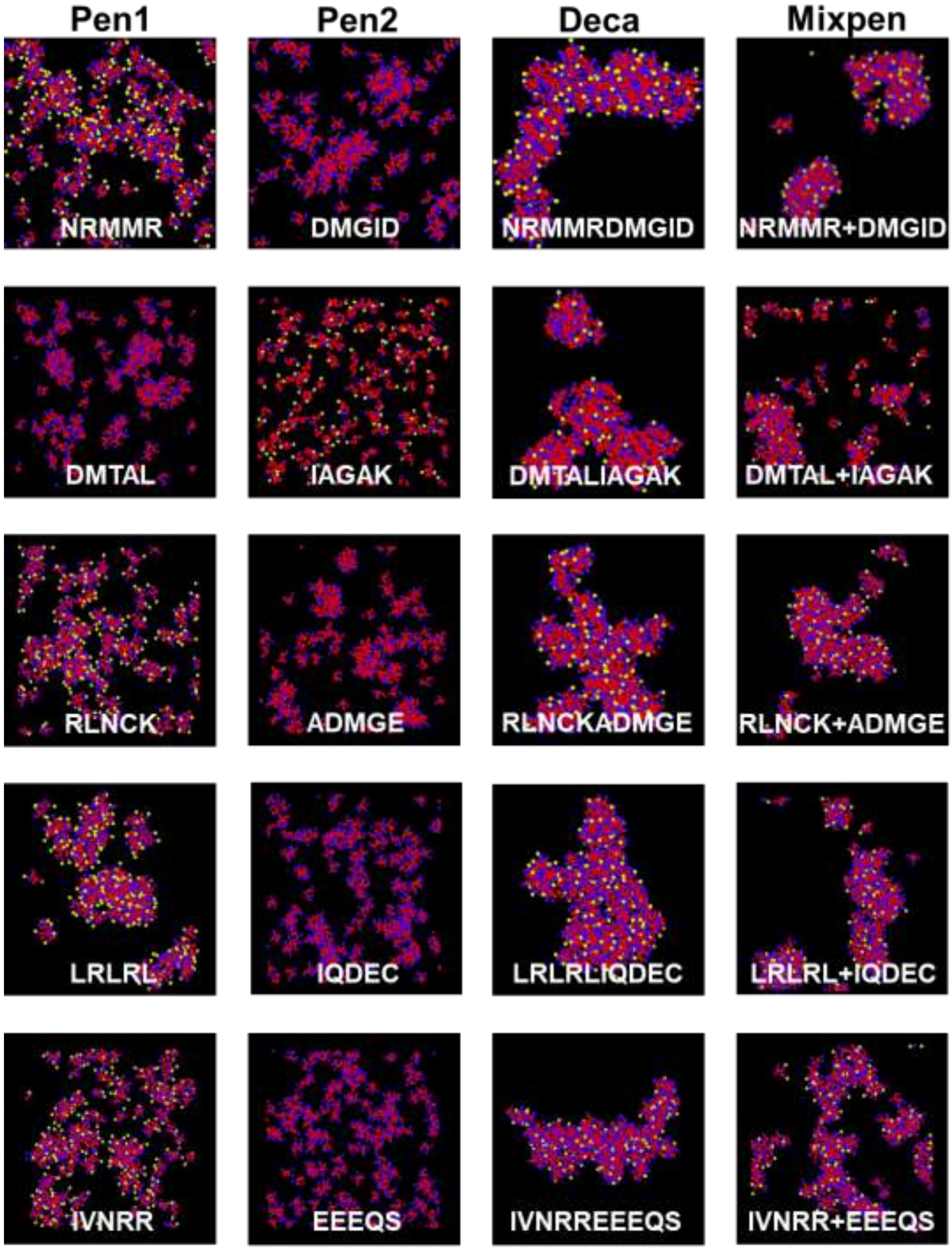

Figure S11. Morphologies of five groups of pentapeptides, decapeptides, and mixed pentapeptides after aggregation for 125 ns.

Supplementary Tables for Main Body

Table S1. Bead representation of 20 natural amino acids. Currently eighteen particle types are defined, divided into four main categories: **P**, polar; **N**, intermediate polar; **C**, apolar; **Q**, charged. Each category has a number of sublevels (**0**, **a**, **d**, or **da**): subtype **0** has no hydrogen bond forming capacities; subtype **a** has some hydrogen acceptor capacities ; subtype **d** has some hydrogen donor capacities; subtype **da** has both donor and acceptor capacities; or (**1**, **2**, **3**, **4**, **5**) where subtype 5 is more polar than 1. **S** indicates the bead is a ring-type bead. Standard beads are mapped using a 4:1 mapping scheme, and **ring** beads which are used for ring compounds are mapped 2-3:1[4]. **N-**, **M**, and **C-** denotes first position close to N-terminus, middle positions from the second to the second last, and last position close to C-terminus, respectively.

	**Amino**	**Structure**	**Coarse-grained structure**			
			**Backbone (BB)**			**Side chain (SC)**
			**N-**	**M**	**C-**	
1	**PHE, F**	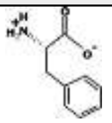	Qd	Nda	Qa	SC5+SC5+SC5
2	**TRP, W**	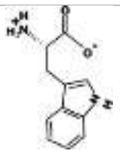	Qd	Nda	Qa	SC4+SNd+SC5+SC5
3	**TYR, Y**	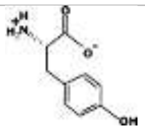	Qd	Nda	Qa	SC4+SC4+SP1
4	**CYS, C**	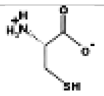	Qd	Nda	Qa	C5
5	**ILE, I**	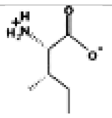	Qd	Nda	Qa	AC1
6	**LEU, L**	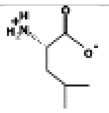	Qd	Nda	Qa	AC1
7	**VAL, V**	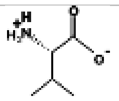	Qd	Nda	Qa	AC2

8	**HIS, H**	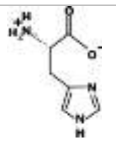	Qd	Nda	Qa	SC4+SP1+SP1
9	**SER, S**	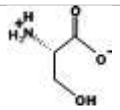	Qd	Nda	Qa	P1
10	**THR, T**	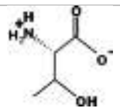	Qd	Nda	Qa	P1
11	**MET, M**	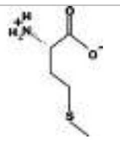	Qd	Nda	Qa	C5
12	**PRO, P**	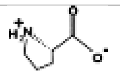	Qd	N0	Qa	C3
13	**ALA, A**	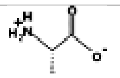	Qd	N0	Qa	-
14	**ASP, D**	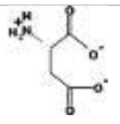	Qd	Nda	Qa	Qa
15	**GLU, E**	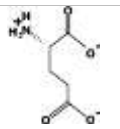	Qd	Nda	Qa	Qa
16	**GLY, G**	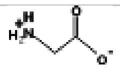	Qd	Nda	Qa	-
17	**LYS, K**	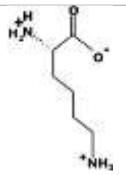	Qd	Nda	Qa	C3+Qd
18	**ASN, N**	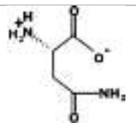	Qd	Nda	Qa	P5
19	**GLN, Q**	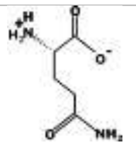	Qd	Nda	Qa	P4
20	**ARG, R**	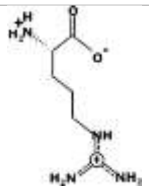	Qd	Nda	Qa	N0+Qd

Table S2. MAE and R^2 of deep-learning (TRN) and five non-deep learning (SVM, RF, NN, BR, LR) AI models trained with 1,000, 5,000, 8,000, and 54,000 data.

Algorithm	Performance	Pep system	#1,000	#5,000	#8,000	#54,000
**TRN** (**T**ransformer-based **R**egression **N**etwork)	**R^2**	Penta	0.78107	0.90579	0.91670	-
		Deca	0.67535	0.82653	0.85179	-
		Mixedpenta	0.73051	0.84889	0.87359	-
		**Combo**	-	-	-	**0.91935**
	**MAE**	Penta	0.10682	0.06973	0.06599	-
		Deca	0.06751	0.04985	0.04695	-
		Mixedpenta	0.07884	0.05956	0.05412	-
		**Combo**	-	-	-	**0.04692**
**SVM** (**S**upport **V**ector **M**achine)	**R^2**	Penta	-	-	0.84700	-
		Deca	-	-	0.76180	-
		Mixedpenta	-	-	0.72446	-
	**MAE**	Penta	-	-	0.08854	-
		Deca	-	-	0.05830	-
		Mixedpenta	-	-	0.07689	-
**RF** (**R**andom **F**orest)	**R^2**	Penta	-	-	0.76240	-
		Deca	-	-	0.55192	-
		Mixedpenta	-	-	0.52683	-
	**MAE**	Penta	-	-	0.10885	-
		Deca	-	-	0.08000	-
		Mixedpenta	-	-	0.09957	-
**NN** (**N**earest **N**eighbor)	**R^2**	Penta	-	-	0.56191	-
		Deca	-	-	0.32296	-
		Mixedpenta	-	-	0.28433	-
	**MAE**	Penta	-	-	0.14829	-
		Deca	-	-	0.09912	-
		Mixedpenta	-	-	0.12262	-
**BR** (**B**ayesian **R**idge)	**R^2**	Penta	-	-	0.77498	-
		Deca	-	-	0.80955	-
		Mixedpenta	-	-	0.73302	-
	**MAE**	Penta	-	-	0.10498	-
		Deca	-	-	0.05132	-
		Mixedpenta	-	-	0.07446	-
**LR** (**L**inear **R**egression)	**R^2**	Penta	-	-	0.77328	-
		Deca	-	-	0.80249	-
		Mixedpenta	-	-	0.72007	-
	**MAE**	Penta	-	-	0.10547	-
		Deca	-	-	0.05248	-
		Mixedpenta	-	-	0.07655	-

Table S3. AP information of decapeptides, corresponding pentapeptides, averaged AP of two pentapeptides, and mixed pentapeptides, with top 200 $AP_{mixpen} – AP_{avepen}$ values. All AP data presented here are predicted by AI models.

**Index**	**Penta1**	**Penta2**	**AP_{penta1}**	**AP_{penta2}**	**AP_{avepen}**	**AP_{deca}**	**AP_{mixpen}**	**AP_{deca}- AP_{avepen}**	**AP_{mixpen}- AP_{avepen}**
1	DEHEW	RKMKC	1.34	1.23	1.29	1.68	1.92	0.39	**0.63**
2	DDEIY	VKKVK	1.16	1.21	1.19	1.62	1.81	0.43	**0.62**
3	SRRPR	EFMED	1.23	1.23	1.23	1.70	1.84	0.47	**0.61**
4	YEESD	RKARP	1.27	1.12	1.19	1.55	1.79	0.36	**0.59**
5	DEEYY	RKISK	1.25	1.31	1.28	1.68	1.87	0.40	**0.59**
6	NKRPK	VDDDW	1.10	1.30	1.20	1.68	1.79	0.49	**0.59**
7	CEEMD	KKRFR	1.04	1.29	1.17	1.58	1.75	0.41	**0.59**
8	PDDDW	YKRKK	1.28	1.25	1.27	1.67	1.85	0.41	**0.59**
9	DDMMD	PKWRR	1.06	1.51	1.29	1.67	1.87	0.38	**0.59**
10	DFEDE	KALKR	1.09	1.17	1.13	1.47	1.72	0.34	**0.59**
11	CRRNR	ELYED	1.17	1.30	1.23	1.67	1.81	0.44	**0.58**
12	PDAEE	YRRKC	0.97	1.39	1.18	1.56	1.76	0.38	**0.58**
13	IKKKC	EIEFM	1.22	1.30	1.26	1.80	1.84	0.54	**0.58**
14	FDEDY	PMRRN	1.28	1.20	1.24	1.66	1.82	0.42	**0.58**
15	KAPRK	DIHDD	1.10	1.34	1.22	1.72	1.80	0.50	**0.57**
16	YEDEH	KTRRM	1.27	1.26	1.27	1.68	1.84	0.41	**0.57**
17	RRWRR	EDVGE	1.36	1.05	1.21	1.64	1.78	0.44	**0.57**
18	YTEED	RRKKT	1.27	1.20	1.24	1.58	1.81	0.34	**0.57**
19	FEESE	TCKRR	1.22	1.25	1.24	1.57	1.80	0.34	**0.57**
20	KKKLA	NEMDF	1.19	1.31	1.25	1.73	1.82	0.48	**0.57**
21	MRIRK	DYDAD	1.20	1.19	1.19	1.68	1.76	0.49	**0.57**
22	DYETI	PLKKK	1.41	1.18	1.29	1.66	1.86	0.36	**0.57**
23	RAKIK	DIESD	1.15	1.14	1.14	1.64	1.71	0.50	**0.57**
24	KVRFR	MFEED	1.44	1.22	1.33	1.77	1.89	0.44	**0.57**
25	RLVKK	DDPVD	1.25	1.07	1.16	1.65	1.72	0.49	**0.57**
26	QEFDE	RARLK	1.25	1.15	1.20	1.55	1.76	0.35	**0.56**
27	EDEIW	RGRKF	1.23	1.37	1.30	1.73	1.87	0.43	**0.56**
28	TKNRR	DFDHE	1.22	1.31	1.26	1.72	1.83	0.45	**0.56**
29	DDHDV	KLGKR	1.22	1.21	1.22	1.57	1.78	0.36	**0.56**
30	LARRI	DDYDL	1.25	1.24	1.24	1.75	1.80	0.51	**0.56**
31	NLKKL	LEDDY	1.28	1.21	1.25	1.71	1.80	0.47	**0.56**
32	APRRV	EYDEY	1.22	1.30	1.26	1.75	1.82	0.50	**0.56**
33	DDVHE	KKTVK	1.26	1.30	1.28	1.58	1.84	0.30	**0.56**
34	KLMKK	TESDE	1.22	1.23	1.23	1.66	1.78	0.43	**0.55**
35	FEDEV	QRCKV	1.15	1.29	1.22	1.58	1.78	0.36	**0.55**
36	SQEEE	IKKTK	1.11	1.25	1.18	1.42	1.73	0.24	**0.55**
37	IEELY	PRKKA	1.40	1.12	1.26	1.63	1.81	0.37	**0.55**
38	VRRNK	EEGYE	1.15	1.18	1.16	1.66	1.71	0.50	**0.55**
39	RRKCS	TAEED	1.32	1.07	1.19	1.62	1.74	0.43	**0.55**
40	RRKYP	CDDDT	1.43	1.13	1.28	1.70	1.83	0.41	**0.55**
41	RTRRK	DEYSD	1.18	1.36	1.27	1.67	1.82	0.40	**0.55**
42	SKKKP	EEGLF	1.24	1.29	1.26	1.73	1.81	0.47	**0.55**

43	WEGDE	NRRRM	1.30	1.14	1.22	1.54	1.76	0.33	**0.55**
44	EFELV	PPCRK	1.33	1.27	1.30	1.69	1.85	0.39	**0.54**
45	QRCQK	DHDEF	1.20	1.29	1.24	1.71	1.79	0.47	**0.54**
46	DPYDE	RGKMP	1.25	1.25	1.25	1.60	1.79	0.35	**0.54**
47	DDMEL	QKVKK	1.02	1.16	1.09	1.35	1.63	0.27	**0.54**
48	VRRQP	ECDEW	1.22	1.27	1.24	1.74	1.79	0.49	**0.54**
49	EEIEW	QVRKG	1.25	1.23	1.24	1.55	1.78	0.31	**0.54**
50	AWRKK	EMDEE	1.36	0.94	1.15	1.62	1.69	0.47	**0.54**
51	DGEMD	MMRKK	1.01	1.14	1.07	1.35	1.61	0.28	**0.54**
52	ARRYK	DDSDA	1.33	1.09	1.21	1.62	1.75	0.41	**0.54**
53	KKLKS	LVDET	1.32	1.27	1.30	1.68	1.84	0.38	**0.54**
54	SDMEF	MPRKV	1.48	1.27	1.37	1.74	1.91	0.37	**0.54**
55	KMMPK	MEEYC	1.28	1.33	1.31	1.84	1.85	0.53	**0.54**
56	MMCRR	QDMEF	1.29	1.29	1.29	1.77	1.83	0.49	**0.54**
57	VRKLK	GMDEF	1.21	1.34	1.27	1.74	1.81	0.47	**0.54**
58	DMDMF	PKYKR	1.34	1.43	1.38	1.76	1.92	0.38	**0.54**
59	DIYDE	ARARK	1.32	1.07	1.19	1.55	1.73	0.36	**0.54**
60	LRAKM	EYLDD	1.22	1.35	1.28	1.72	1.81	0.44	**0.53**
61	RPRMN	YVEDD	1.21	1.26	1.24	1.71	1.77	0.47	**0.53**
62	RKKVF	EEMYP	1.50	1.33	1.41	1.84	1.95	0.42	**0.53**
63	MPRCK	EIEYC	1.25	1.33	1.29	1.83	1.83	0.53	**0.53**
64	DECHN	RIMKK	1.34	1.21	1.28	1.61	1.81	0.33	**0.53**
65	SELDP	HMKKK	1.28	1.33	1.30	1.66	1.83	0.36	**0.53**
66	MCDDC	RKMYK	1.22	1.45	1.34	1.75	1.87	0.41	**0.53**
67	MKKKH	MQDES	1.39	1.18	1.29	1.66	1.82	0.38	**0.53**
68	KRKVV	EEHYF	1.25	1.69	1.47	1.89	2.00	0.42	**0.53**
69	PDEVL	KRCKI	1.17	1.26	1.22	1.61	1.75	0.40	**0.53**
70	PVKRR	PDPEY	1.18	1.38	1.28	1.74	1.81	0.47	**0.53**
71	SDDLL	MKMKK	1.33	1.16	1.24	1.60	1.77	0.36	**0.53**
72	DSEML	IQKKK	1.18	1.13	1.15	1.49	1.68	0.33	**0.53**
73	AKKIK	HEQLD	1.16	1.38	1.27	1.65	1.80	0.38	**0.53**
74	DLMDM	KKMFK	1.18	1.49	1.33	1.74	1.86	0.41	**0.53**
75	KTKKC	DFEVI	1.28	1.37	1.33	1.76	1.86	0.43	**0.53**
76	CKRGP	IDPEH	1.26	1.34	1.30	1.71	1.82	0.42	**0.53**
77	TEEHA	PRKMA	1.35	1.21	1.28	1.63	1.81	0.34	**0.53**
78	SGLKK	EDYEC	1.37	1.20	1.28	1.70	1.81	0.42	**0.53**
79	EYDDC	LRMKM	1.22	1.32	1.27	1.67	1.80	0.40	**0.53**
80	SKVRR	DDDNF	1.28	1.14	1.21	1.66	1.74	0.45	**0.53**
81	GKRKH	LDHED	1.39	1.30	1.35	1.67	1.87	0.33	**0.53**
82	DMDVY	QVKKV	1.37	1.27	1.32	1.70	1.85	0.38	**0.52**
83	PRCKA	DELLH	1.26	1.38	1.32	1.79	1.85	0.47	**0.52**
84	HPDDP	PRKMC	1.36	1.30	1.33	1.73	1.85	0.40	**0.52**
85	YECDL	LPKKN	1.43	1.22	1.32	1.67	1.84	0.35	**0.52**
86	KMRRY	MEHDM	1.41	1.33	1.37	1.80	1.89	0.43	**0.52**
87	IIEEV	KKPKS	1.24	1.27	1.25	1.62	1.78	0.37	**0.52**
88	ECESD	YKRRS	1.13	1.49	1.31	1.62	1.83	0.31	**0.52**
89	ALKRL	DPEWV	1.29	1.44	1.36	1.79	1.89	0.43	**0.52**
90	VDDDW	RGMKL	1.30	1.31	1.31	1.69	1.83	0.38	**0.52**
91	ICCRR	EFCDD	1.37	1.30	1.33	1.75	1.85	0.42	**0.52**

92	ELDEY	PKTRP	1.19	1.35	1.27	1.65	1.79	0.38	**0.52**
93	PDDES	KRRSR	1.09	1.22	1.15	1.47	1.67	0.31	**0.52**
94	DEHPP	PRMTK	1.29	1.34	1.31	1.68	1.83	0.36	**0.52**
95	WIRRR	DDDTC	1.43	1.09	1.26	1.70	1.78	0.44	**0.52**
96	KIHKK	EFEHE	1.50	1.27	1.39	1.80	1.91	0.41	**0.52**
97	AKKCK	EDEVD	1.15	0.97	1.06	1.56	1.58	0.50	**0.52**
98	IKAKP	DDYIE	1.22	1.29	1.25	1.74	1.77	0.48	**0.52**
99	KRPRN	LDTED	1.16	1.18	1.17	1.63	1.69	0.47	**0.52**
100	MRNKV	EDHSD	1.23	1.37	1.30	1.69	1.82	0.39	**0.52**
101	VYDDE	NTRKP	1.28	1.29	1.29	1.57	1.80	0.28	**0.52**
102	HEPDH	IRKRV	1.44	1.22	1.33	1.69	1.85	0.36	**0.52**
103	DEPQY	LRKMM	1.21	1.31	1.26	1.66	1.78	0.40	**0.52**
104	NWRKK	MEEYV	1.35	1.32	1.34	1.77	1.85	0.43	**0.52**
105	WEEEC	VKMVR	1.24	1.37	1.30	1.66	1.82	0.36	**0.52**
106	DWDTD	LKRAR	1.42	1.13	1.27	1.61	1.79	0.33	**0.52**
107	DLMCD	RKKIV	1.30	1.25	1.28	1.60	1.79	0.33	**0.52**
108	RRLLR	HEEFQ	1.27	1.37	1.32	1.76	1.84	0.44	**0.52**
109	VAKPR	DEPVH	1.18	1.30	1.24	1.76	1.76	0.52	**0.52**
110	RHLRR	EEYDG	1.49	1.17	1.33	1.72	1.85	0.39	**0.52**
111	ADMEF	RPRRL	1.30	1.20	1.25	1.60	1.76	0.35	**0.52**
112	KRKPW	DAEWP	1.51	1.30	1.41	1.81	1.92	0.41	**0.51**
113	FIDEE	ALLKK	1.23	1.31	1.27	1.64	1.78	0.37	**0.51**
114	KKKLL	QEQCD	1.26	1.14	1.20	1.63	1.72	0.43	**0.51**
115	VIKIR	DFEDS	1.41	1.25	1.33	1.74	1.84	0.42	**0.51**
116	EDEYS	KRGKY	1.22	1.43	1.32	1.68	1.84	0.36	**0.51**
117	AEEDY	QPKKI	1.14	1.22	1.18	1.50	1.70	0.32	**0.51**
118	IHEAD	ALRKL	1.33	1.29	1.31	1.66	1.82	0.36	**0.51**
119	FRKVR	EEFMP	1.37	1.37	1.37	1.82	1.88	0.45	**0.51**
120	DMENY	KPKKM	1.26	1.16	1.21	1.61	1.72	0.40	**0.51**
121	TQRRK	NTDED	1.19	1.17	1.18	1.60	1.69	0.42	**0.51**
122	HDDDW	RPIPK	1.38	1.33	1.35	1.70	1.87	0.35	**0.51**
123	RMRVV	LAEEY	1.38	1.27	1.33	1.76	1.84	0.43	**0.51**
124	EEEWV	RRLNI	1.22	1.34	1.28	1.62	1.79	0.34	**0.51**
125	PNDED	KKPVK	1.05	1.21	1.13	1.37	1.64	0.24	**0.51**
126	FMDEI	IAKKM	1.44	1.22	1.33	1.75	1.84	0.42	**0.51**
127	MRFRK	DAPEL	1.35	1.04	1.19	1.67	1.70	0.47	**0.51**
128	DCDYQ	TPKPK	1.29	1.27	1.28	1.64	1.79	0.37	**0.51**
129	HDTDD	MYRKR	1.36	1.34	1.35	1.68	1.86	0.32	**0.51**
130	TRMAK	YIEDD	1.26	1.27	1.27	1.69	1.78	0.43	**0.51**
131	SEDAD	RKKTR	1.09	1.20	1.15	1.40	1.66	0.26	**0.51**
132	PDFDQ	CPKKI	1.42	1.32	1.37	1.72	1.88	0.35	**0.51**
133	EHDVV	KKSCR	1.35	1.31	1.33	1.66	1.84	0.33	**0.51**
134	DPMDS	YKRKT	1.20	1.45	1.33	1.68	1.84	0.36	**0.51**
135	LRYKK	EDQLT	1.42	1.15	1.28	1.68	1.79	0.39	**0.51**
136	CDDEV	RGLRR	1.05	1.21	1.13	1.47	1.64	0.34	**0.51**
137	RCRFR	EACDY	1.43	1.28	1.35	1.79	1.86	0.44	**0.51**
138	TRKPA	PECDY	1.27	1.42	1.34	1.78	1.85	0.43	**0.51**
139	MQDDE	RKWRC	1.03	1.61	1.32	1.63	1.83	0.32	**0.51**
140	RTLKA	TEEMD	1.40	1.09	1.25	1.64	1.75	0.39	**0.51**

141	RQRVV	EQEFY	1.30	1.36	1.33	1.80	1.84	0.47	**0.51**
142	MDCVE	RIKFK	1.27	1.42	1.35	1.70	1.85	0.35	**0.51**
143	NDDFD	LRNCR	1.27	1.26	1.27	1.56	1.77	0.29	**0.51**
144	KAKLI	EMDHL	1.33	1.31	1.32	1.79	1.83	0.47	**0.51**
145	RKTCK	DFWED	1.29	1.61	1.45	1.79	1.96	0.34	**0.51**
146	DCEVY	SLKKQ	1.37	1.33	1.35	1.66	1.85	0.31	**0.51**
147	EVEFA	CVKVK	1.26	1.35	1.30	1.70	1.81	0.39	**0.51**
148	IEEHQ	KIPNK	1.26	1.25	1.26	1.60	1.76	0.35	**0.51**
149	LSEDM	CRKCC	1.23	1.37	1.30	1.66	1.80	0.36	**0.51**
150	YCEDE	NMKLK	1.23	1.22	1.22	1.54	1.73	0.32	**0.51**
151	AMRKM	ELHDP	1.21	1.43	1.32	1.74	1.83	0.42	**0.51**
152	VECEY	GRCKK	1.38	1.18	1.28	1.62	1.78	0.35	**0.51**
153	EFEGF	CVKRM	1.46	1.31	1.39	1.76	1.89	0.37	**0.51**
154	VVKKI	EMEAF	1.42	1.21	1.32	1.80	1.82	0.48	**0.51**
155	EEGTE	IRKKP	1.08	1.18	1.13	1.39	1.64	0.26	**0.51**
156	YAELE	VGKMR	1.29	1.25	1.27	1.62	1.78	0.35	**0.51**
157	ACKLK	VEDNF	1.27	1.29	1.28	1.69	1.79	0.41	**0.51**
158	WEDLD	PCRRG	1.35	1.26	1.31	1.65	1.81	0.35	**0.51**
159	KVEKK	WEDLD	1.27	1.35	1.31	1.72	1.81	0.41	**0.51**
160	SIRKK	PQDEW	1.25	1.43	1.34	1.72	1.84	0.39	**0.50**
161	YDNDE	CNKMR	1.23	1.21	1.22	1.47	1.72	0.25	**0.50**
162	ELECF	MKRIA	1.39	1.25	1.32	1.71	1.82	0.39	**0.50**
163	ITDEH	IRRLK	1.46	1.21	1.34	1.68	1.84	0.35	**0.50**
164	AVDDM	KFRLK	1.15	1.41	1.28	1.66	1.78	0.38	**0.50**
165	HFKKK	INEDV	1.52	1.14	1.33	1.71	1.84	0.38	**0.50**
166	DVDYQ	PKRCT	1.29	1.41	1.35	1.67	1.85	0.32	**0.50**
167	QKCRV	FDEQH	1.29	1.34	1.32	1.74	1.82	0.42	**0.50**
168	FEENH	NKKLC	1.36	1.28	1.32	1.68	1.83	0.36	**0.50**
169	KQRKC	VQEEE	1.17	1.02	1.10	1.59	1.60	0.49	**0.50**
170	ADFEV	KIKNV	1.39	1.31	1.35	1.63	1.85	0.27	**0.50**
171	VARKP	EPMDF	1.20	1.38	1.29	1.80	1.79	0.51	**0.50**
172	LNEEF	KIRQP	1.30	1.26	1.28	1.63	1.78	0.35	**0.50**
173	DDYWE	LARMK	1.58	1.20	1.39	1.70	1.89	0.31	**0.50**
174	DYEEP	QKRSP	1.15	1.31	1.23	1.58	1.73	0.35	**0.50**
175	FEYED	TRALR	1.40	1.29	1.35	1.65	1.85	0.30	**0.50**
176	RQMKP	EWEPV	1.23	1.41	1.32	1.80	1.82	0.48	**0.50**
177	KKSKV	LDDGC	1.30	1.18	1.24	1.64	1.74	0.40	**0.50**
178	IRLRN	NDMEY	1.30	1.32	1.31	1.75	1.81	0.44	**0.50**
179	WEEEL	KPCGR	1.24	1.28	1.26	1.62	1.76	0.36	**0.50**
180	RKKYQ	DFEIP	1.39	1.33	1.36	1.81	1.86	0.44	**0.50**
181	FNMKR	QDDCC	1.44	1.15	1.29	1.68	1.80	0.39	**0.50**
182	KRKLY	DDVVQ	1.52	1.13	1.33	1.70	1.83	0.37	**0.50**
183	MASDD	MKCKN	1.27	1.27	1.27	1.52	1.77	0.25	**0.50**
184	DDIEF	KIQKS	1.20	1.38	1.29	1.60	1.79	0.31	**0.50**
185	DECVY	KVQKV	1.39	1.29	1.34	1.67	1.84	0.33	**0.50**
186	EAEFE	RRKRN	1.11	1.12	1.11	1.39	1.61	0.28	**0.50**
187	FRYRK	DIDPA	1.59	1.08	1.34	1.71	1.83	0.38	**0.50**
188	AEEFP	MCVRK	1.26	1.32	1.29	1.67	1.79	0.38	**0.50**
189	QRCQR	DIFDD	1.21	1.41	1.31	1.66	1.81	0.35	**0.50**

190	IPVED	KRKKW	1.32	1.36	1.34	1.67	1.84	0.33	**0.50**
191	CTKRP	ISEVD	1.34	1.32	1.33	1.67	1.83	0.34	**0.50**
192	EGDVH	RMCKA	1.30	1.29	1.30	1.63	1.79	0.33	**0.50**
193	ELDHS	NRKAP	1.41	1.13	1.27	1.61	1.77	0.35	**0.50**
194	RPGRV	DNFDD	1.27	1.30	1.29	1.69	1.79	0.40	**0.50**
195	IRKPC	DLLDS	1.32	1.35	1.34	1.73	1.83	0.39	**0.50**
196	SEDEH	IGKTK	1.26	1.33	1.29	1.59	1.79	0.29	**0.50**
197	RIRKN	EEGYY	1.18	1.45	1.32	1.77	1.82	0.46	**0.50**
198	KHYKK	PIDEC	1.69	1.21	1.45	1.77	1.95	0.32	**0.50**
199	CRMVK	DQYDM	1.34	1.32	1.33	1.78	1.83	0.45	**0.50**
200	RRVMN	EFMEV	1.30	1.36	1.33	1.78	1.83	0.45	**0.50**

Table S4. AP information of decapeptides, corresponding pentapeptides, averaged AP of two pentapeptides, and mixed pentapeptides, with top 100 AP_{mixpen} – AP_{avepen} values. All AP data presented here are generated by CGMD simulations.

Index	Penta1	Penta2	AP_{penta1}	AP_{penta2}	AP_{avepen}	AP_{deca}	AP_{mixpen}	AP_{deca}- AP_{avepen}	AP_{mixpen}- AP_{avepen}
**1**	YHDDC	NRGMR	1.61	1.12	1.36	1.73	1.85	0.37	**0.49**
**2**	NRMMR	DMGID	1.15	1.14	1.15	1.66	1.61	0.51	**0.47**
**3**	RLNCK	ADMGE	1.17	1.13	1.15	1.59	1.61	0.45	**0.46**
**4**	LRLRL	IQDEC	1.48	1.10	1.29	1.73	1.74	0.44	**0.44**
**5**	NKIMR	PYAEE	1.29	1.47	1.38	1.74	1.82	0.36	**0.44**
**6**	MRRLI	NDCDG	1.45	1.11	1.28	1.69	1.72	0.41	**0.43**
**7**	FGHHE	CMKNK	1.83	1.12	1.48	1.78	1.91	0.31	**0.43**
**8**	HFEFT	TQAGK	1.96	1.15	1.55	1.72	1.98	0.17	**0.43**
**9**	DMSEN	KRRDR	1.19	1.22	1.21	1.42	1.63	0.21	**0.43**
**10**	LEKKP	NEDMW	1.32	1.49	1.40	1.73	1.82	0.32	**0.42**
**11**	ALSRK	DFWEQ	1.34	1.63	1.48	1.71	1.88	0.22	**0.40**
**12**	QHWED	LARRT	1.76	1.21	1.49	1.74	1.88	0.26	**0.40**
**13**	DVADK	RRWRD	1.09	1.73	1.41	1.62	1.80	0.21	**0.39**
**14**	TFQHW	KACAN	2.07	1.19	1.63	1.79	2.02	0.16	**0.39**
**15**	IVNRR	EEEQS	1.17	1.04	1.10	1.53	1.49	0.43	**0.39**
**16**	HWCEM	LVKGK	1.86	1.22	1.54	1.83	1.92	0.29	**0.38**
**17**	RKTQP	EGVHE	1.40	1.56	1.48	1.71	1.85	0.24	**0.37**
**18**	WRKRF	ADTDH	1.61	1.51	1.56	1.78	1.92	0.22	**0.36**
**19**	KCKVC	QEMDE	1.53	1.05	1.29	1.62	1.64	0.33	**0.35**
**20**	LDEHD	LNAIR	1.21	1.39	1.30	1.46	1.65	0.16	**0.35**
**21**	NRFKP	QAENH	1.61	1.33	1.47	1.70	1.81	0.23	**0.34**
**22**	MLRYK	EYMPC	1.67	1.82	1.74	1.89	2.08	0.15	**0.34**
**23**	FEYQG	YAMVK	1.67	1.73	1.70	1.83	2.04	0.13	**0.34**
**24**	AVFYQ	CAEIM	1.99	1.14	1.57	1.80	1.91	0.23	**0.34**
**25**	EPWTL	MQRKT	1.85	1.21	1.53	1.73	1.87	0.20	**0.34**
**26**	SLVDD	FERKK	1.63	1.40	1.51	1.71	1.85	0.19	**0.34**
**27**	CVRNK	EYDRW	1.18	1.85	1.52	1.76	1.85	0.25	**0.34**
**28**	RALYW	AADEP	1.98	1.01	1.50	1.84	1.83	0.34	**0.33**
**29**	IQIAK	TNETW	1.38	1.78	1.58	1.75	1.91	0.16	**0.33**
**30**	NCRLK	HLLDL	1.16	1.76	1.46	1.69	1.79	0.23	**0.33**
**31**	MAVRK	AHDCA	1.14	1.54	1.34	1.67	1.67	0.33	**0.32**
**32**	CVWEE	AKSKN	1.71	1.36	1.53	1.66	1.86	0.13	**0.32**
**33**	CSKMK	DKEYC	1.30	1.62	1.46	1.69	1.79	0.23	**0.32**
**34**	ITEIG	ISRKK	1.56	1.16	1.36	1.53	1.68	0.17	**0.32**
**35**	YRVVK	PGEWE	1.67	1.65	1.66	1.84	1.98	0.18	**0.32**
**36**	VRLWM	VEHEY	2.01	1.41	1.71	1.99	2.03	0.27	**0.31**
**37**	FEVEP	GRCWP	1.25	1.88	1.56	1.80	1.88	0.23	**0.31**
**38**	EFYLH	CRMRK	2.06	1.12	1.59	1.76	1.90	0.17	**0.31**

**39**	PADVQ	SANFR	1.07	1.78	1.42	1.69	1.73	0.26	**0.31**
**40**	YVWVT	KPRGI	2.22	1.20	1.71	1.86	2.02	0.15	**0.31**
**41**	QGCAI	FARRY	1.30	1.69	1.49	1.65	1.80	0.15	**0.31**
**42**	ETWNG	RLKVK	1.75	1.15	1.45	1.61	1.76	0.16	**0.31**
**43**	MLRQG	SGYYF	1.24	2.37	1.81	2.01	2.11	0.21	**0.31**
**44**	PCFKS	EPNIP	2.03	1.04	1.54	1.82	1.84	0.29	**0.31**
**45**	IAGRP	YSSYS	1.25	1.98	1.61	1.85	1.92	0.23	**0.30**
**46**	PVRLA	TIYFD	1.61	2.09	1.85	1.97	2.15	0.13	**0.30**
**47**	DGNCF	WKTRN	1.52	1.64	1.58	1.65	1.88	0.07	**0.30**
**48**	QVKYT	ADINP	1.93	1.23	1.58	1.71	1.88	0.13	**0.30**
**49**	DCYQE	DGQKR	1.47	1.19	1.33	1.53	1.63	0.20	**0.30**
**50**	SIPKA	DECIP	1.65	1.06	1.35	1.70	1.65	0.35	**0.30**
**51**	MDMED	KMCLW	1.07	1.98	1.52	1.71	1.82	0.19	**0.30**
**52**	KQKLN	IEGMN	1.19	1.16	1.17	1.44	1.47	0.27	**0.30**
**53**	TQDYF	RVAKC	1.98	1.28	1.63	1.77	1.93	0.14	**0.30**
**54**	VLKYR	HEIMD	1.72	1.60	1.66	1.78	1.96	0.12	**0.30**
**55**	WFFHH	FLEGC	2.09	1.79	1.94	2.02	2.24	0.09	**0.30**
**56**	KGALP	AWETW	1.50	1.96	1.73	1.84	2.03	0.11	**0.30**
**57**	WDNAC	SMRKG	1.68	1.30	1.49	1.63	1.79	0.14	**0.30**
**58**	GWKFH	DVNLY	1.95	1.79	1.87	1.93	2.16	0.06	**0.30**
**59**	KMNPT	FEFTL	1.43	2.02	1.72	1.86	2.02	0.14	**0.29**
**60**	REWCR	ETALD	1.86	1.10	1.48	1.71	1.78	0.23	**0.29**
**61**	KKIIL	ENALA	1.63	1.04	1.34	1.66	1.63	0.33	**0.29**
**62**	IIWDW	LEMKK	2.22	1.37	1.79	1.85	2.09	0.05	**0.29**
**63**	DPWDV	RRAVW	1.67	1.69	1.68	1.85	1.97	0.16	**0.29**
**64**	RLWMV	HEIQN	1.89	1.41	1.65	1.75	1.94	0.10	**0.29**
**65**	PIEVG	GYLKI	1.46	1.81	1.63	1.80	1.92	0.17	**0.29**
**66**	MHWGW	CMDDV	2.18	1.16	1.67	1.83	1.96	0.16	**0.29**
**67**	VVRML	NWDIQ	1.79	1.67	1.73	1.81	2.02	0.08	**0.29**
**68**	WGICR	PDMLG	1.87	1.48	1.68	1.82	1.96	0.15	**0.29**
**69**	VTHLH	AGRPQ	2.01	1.10	1.55	1.73	1.84	0.18	**0.29**
**70**	SHDVD	NVKPP	1.66	1.43	1.54	1.66	1.83	0.12	**0.29**
**71**	GFIEQ	IRYRV	1.62	1.72	1.67	1.75	1.95	0.08	**0.28**
**72**	EHCWT	GRKMS	1.93	1.41	1.67	1.75	1.95	0.08	**0.28**
**73**	LQCLR	DIFDG	1.59	1.53	1.56	1.72	1.84	0.17	**0.28**
**74**	YYWYH	RTMAN	2.21	1.48	1.84	1.88	2.12	0.04	**0.28**
**75**	GECQC	NPVRR	1.12	1.18	1.15	1.42	1.43	0.28	**0.28**
**76**	TDNML	RSIKH	1.53	1.72	1.63	1.70	1.91	0.07	**0.28**
**77**	VQTFH	MDVNA	1.92	1.17	1.55	1.76	1.83	0.21	**0.28**
**78**	TEDYQ	CCMRA	1.47	1.61	1.54	1.71	1.82	0.17	**0.28**
**79**	ASEDV	GNHRM	1.20	1.72	1.46	1.57	1.74	0.11	**0.28**
**80**	WEYEM	IAGVK	1.61	1.45	1.53	1.78	1.81	0.25	**0.28**
**81**	QNRCY	DDHDH	1.71	1.35	1.53	1.71	1.80	0.19	**0.28**
**82**	VIWEW	VCKQA	2.25	1.34	1.80	1.89	2.07	0.09	**0.28**
**83**	GSDLF	PIKQR	1.77	1.17	1.47	1.67	1.74	0.20	**0.27**
**84**	ECTVD	DYRAR	1.34	1.58	1.46	1.60	1.73	0.14	**0.27**
**85**	HFVKD	STPEM	1.92	1.53	1.73	1.78	2.00	0.05	**0.27**
**86**	DMTAL	IAGAK	1.16	1.03	1.09	1.54	1.37	0.45	**0.27**

**87**	VPNAE	MYHSS	1.10	2.07	1.58	1.78	1.86	0.20	**0.27**
**88**	DICRK	WLDMM	1.40	1.91	1.65	1.81	1.92	0.15	**0.27**
**89**	PLEVN	KPQSW	1.21	2.02	1.62	1.80	1.89	0.19	**0.27**
**90**	YVRWH	LDCVE	2.05	1.40	1.73	1.86	2.00	0.14	**0.27**
**91**	FRCHN	SIEEC	1.97	1.29	1.63	1.78	1.90	0.15	**0.27**
**92**	SWKCY	QDQHP	2.11	1.44	1.77	1.86	2.04	0.09	**0.27**
**93**	SGWMC	EPCRR	2.05	1.20	1.62	1.77	1.89	0.15	**0.27**
**94**	VTMAN	CQHTC	1.60	1.84	1.72	1.77	1.98	0.06	**0.27**
**95**	KKITN	CQAEY	1.48	1.51	1.49	1.75	1.76	0.26	**0.27**
**96**	WQECP	GKKNC	1.87	1.17	1.52	1.70	1.78	0.18	**0.26**
**97**	ANRRI	CTIHD	1.16	1.94	1.55	1.72	1.82	0.17	**0.26**
**98**	HLWVF	AIRMI	2.26	1.69	1.98	2.01	2.24	0.03	**0.26**
**99**	HVWCT	DAKQM	2.01	1.14	1.57	1.83	1.83	0.26	**0.26**
**100**	FRFQH	EQRAI	1.97	1.10	1.54	1.72	1.80	0.19	**0.26**

Supplementary References

1. Laskowski RA, MacArthur MW, Moss DS, Thornton JM. PROCHECK: a program to check the stereochemical quality of protein structures. *J. Appl. Crystallogr.* **26**, 283-291 (1993).

2. Hovmöller S, Zhou T, Ohlson T. Conformations of amino acids in proteins. *Acta Crystallogr., Sect. D: Biol. Crystallogr.* **58**, 768-776 (2002).

3. Berendsen HJ, van der Spoel D, van Drunen R. GROMACS: A message-passing parallel molecular dynamics implementation. *Comp. Phys. Commun.* **91**, 43-56 (1995).

4. Marrink SJ, Risselada HJ, Yefimov S, Tieleman DP, De Vries AH. The MARTINI force field: coarse grained model for biomolecular simulations. *J. Phys. Chem. B* **111**, 7812-7824 (2007).

5. Wang J, Liu K, Xing R, Yan X. Peptide self-assembly: thermodynamics and kinetics. *Chem. Soc. Rev.* **45**, 5589-5604 (2016).

6. Li J, Du X, Hashim S, Shy A, Xu B. Aromatic–aromatic interactions enable α-Helix to β-sheet transition of peptides to form supramolecular hydrogels. *J. Am. Chem. Soc.* **139**, 71-74 (2017).

7. Frederix PW, *et al.* Exploring the sequence space for (tri-) peptide self-assembly to design and discover new hydrogels. *Nat. Chem.* **7**, 30-37 (2015).

8. White SH, Wimley WC. Hydrophobic interactions of peptides with membrane interfaces. *Biochim. Biophys. Acta, Rev. Biomembr.* **1376**, 339-352 (1998).

9. Batra R, *et al.* Machine learning overcomes human bias in the discovery of self-assembling peptides. *Nat. Chem.* 1-9 (2022).

10. Chou PY, Fasman GD. Prediction of protein conformation. *Biochemistry* **13**, 222-245 (1974).
